
JOURNAL INFORMATION
==============================
NLM Title Abbreviation: Med Chir Rev
NLM Journal ID: 0034617

The Medico-Chirurgical Review

Publisher: Biomedical Journal Digitization Project

ARTICLE INFORMATION
==============================
PMCID: PMC4966250
PMID: 29917622
Article version: 1
Subjects: Articles

Hospital Practice

Print publication date: 1828 Oct 1
Volume: 9
Issue: 18
First page: 490
Last page: 525
1828 Aug
License: This work is free of known copyright restrictions. For more information, please see PMC Back Issue Digitization.
License URL: https://creativecommons.org/publicdomain/mark/1.0/

==============================

in one individual who was drowned; a fact also observed by Lafosse, and more recently by M. Avissard.

"7. The dissection of more than fifty persons who had been drowned has satisfied M. Orfila that it is a mistake to suppose that in drowning the individuals die during inspiration, and that, in consequence, they have the diaphragm pushed into the abdomen, and the chest elevated.

"8. He regards the colour of the abdominal viscera as indicative of asphyxia in general, but not of drowning in particular.

"9. The experiments of Orfila, and some other physiologists, show that water enters the stomachs of those who drown themselves; while this is not the case with regard to bodies thrown into the water. But, in order to give its full value to this sign, it would require to be proved that the water had neither been swallowed before death, nor injected after it.

"10. It is not true that the epiglottis is pushed down upon the larynx.

"11. Great importance had been attributed to the presence of sanguineous froth in the windpipe. M. Orfila objects, however, that this exists in other cases, as in death from epilepsy and hanging; while it is wanting in the drowned who have remained long under water, without coming to the surface to breathe.

"M. Orfila made a considerable number of experiments to ascertain if water enters into the ramifications of the bronchia of the drowned; and he has always found that the fluid entered the lungs of the animal, and that the quantity was greater or less in proportion to the care took to keep the head erect in lifting the animal out of the water. But the fluid is not found in the air-passages, if the examination be made soon after death. As to the question, whether the water enters the lungs after death, the experiments of M. Orfila agree with those of M. Piorry, proving that it does enter, and more completely the more vertical the position of the animal; and consequently, the presence of water is not a sufficient proof of drowning, as water enters the air-passages of the dead body, and that the only absolute sign is the presence of water in the minute ramifications of the bronchia; it being proved that this water is the same as that in which the individual was immersed, that it had been injected after death, and could not have penetrated to the lungs from the vertical position of the body in the liquid."

---

### HOSPITAL PRACTICE.

### 45. ABSCESS IN THE LUNGS AFTER INJURIES AND OPERATIONS.

[St. George's Hospital.]

Prior to the publication of Mr. Rose's paper in the Medico-Chirurgical Transactions, the attention of the profession in this country was but little directed to the above very fatal affection. It is true that Mr. Guthrie and Mr. Bell had previously attended to it, and that its frequency and importance had been appreciated and perhaps overrated by Dessault upon the Continent. These notices, however, were diffused in elaborate works, and quite beyond the reach of the majority of those, who become acquainted by means of the medical journals, with the transactions of medical societies. Mr. Rose's paper, or an abstract of it, will therefore be read where the works of Mr. Bell or Mr. Guthrie most probably will not, and with the view of contributing some further illustrations, we intend to bring together some cases which have recently occurred.

We alluded, in a previous article, to the frequent, we might almost say constant, occurrence of pulmonary abscesses or pleural inflammation, as a sequence or consequence of phlebitis. Cavil as we may on the why and the wherefore, the fact is undeniable, and as we have dwelt upon the subject already, we pursue it no further at present.

The case which we are going to detail occurred very recently at St. George's, and, as usually happens, the affection of the lungs proceeded to a fatal termination, with scarcely a symptom to indicate its presence.

*Case* 1.—*Compound Fracture of the Right Leg.*—*Abscesses in the Lungs and Liver.* Robert Rich, ætatis 37, a healthy looking muscular man, was admitted into hospital, 23d of June, under the care of Mr. Keate, with compound fracture of the tibia and fibula, a little below their centre. The external wound was of no great size, but considerable effusion had taken place underneath the integuments, and the acromio-clavicular articulation had been injured, but apparently neither fractured nor displaced. The injury was received in falling from a cart, when a sack of coals came down upon the leg.

The limb was placed in junks, and lint and adhesive plaster applied upon the wound. Spirit lotion—haustus anodynus—haustus sennæ.

On the 26th he was feverish, and ordered infusion of roses, with dilute sulphuric acid and sulphate of magnesia. On the 30th, he had a rigor, succeeded by head-ache, and pyrexia. Castor oil, the liquor opii sedativus, and camphor mixture were employed, and, on the 2d of July, he was almost free from fever, the pulse had subsided, the tongue was cleaning, the bowels were open, and matter appeared to be forming round the broken extremities of the bones.

He continued so well, that the limb, on the 4th, was put up in Mr. Amesbury's apparatus, and every thing appeared to be favourable till the evening of the 9th. On that day he complained of some head-ache, and, next morning, was seized with a rigor, which was followed by heat and malaise. The tongue was whitish—the pulse was full and 80.

*Syr. papav.* ʒj.—*Mist. camph.* ℥iss. *ter die.*

The rigor returned in the night, and again on the morning of the 11th, accompanied with nausea, head-ache, and frequency of pulse. The leg was not painful.

*Sod. carb.* ℈j.—*Magnes. carb.* grs. vj. *Pulv. rhei,* grs. ij.—*T. opii,* ♏. v.—*Mist. camph.* ℥iss. *6ta. quâque horâ.*—*Cataplasma panis cruri.*

The rigors were frequent in the course of the 11th, and attended with a good deal of tenderness in the epigastrium and right hypochondrium. The vomiting was constant both of food and medicine—the tongue was brown—the pulse 120 and fluttering. He was ordered an ounce of camphor mixture, and a little alteration was made in the draught. The shivering returned in the night, but he was better, notwithstanding, on the 12th; the tenderness of the epigastrium was diminished; the tongue was not so brown; the pulse was no more than 100 and firmer in its beat; and the vomiting had somewhat subsided.

The medicines were repeated, but the improvement of the 12th was fallacious, for he was seized with delirium in the night, and on the 13th was worse than before. The pulse was irregular, and had risen to 125—he vomited every thing he took, and lay in a condition approaching to coma, except that he was sensible when roused.

A draught, containing two grains of quinine, four minims of dilute sulphuric acid, half a scruple of aromatic confection, and ten drams of camphor mixture, was given twice a day, with the addition at night of an ounce of æther mixture, and forty minims of tincture of opium. On the 14th he was apparently better; but there was still much abdominal tenderness. On the 15th the symptoms were aggravated; pulse small and 120—vomiting—delirium—tenderness of abdomen. Twenty leeches were applied to the belly, but without effect. The abdomen grew tympanitic—the pulse more rapid—he vomited every thing—was delirious, and on the evening of the 17th he died.

We should state that the liquor ammoniæ and a blister had been applied to the abdomen the day before his death; the discharge from the wound, too, had almost entirely ceased.

*Sectio Cadaveris.* Some opacity of the tunica arachnoides, especially at the base of the brain, and slight depositions of lymph. The plexus choroides presented a singular appearance, being converted into a gland-like tubercular body.

The principal disease was in the thorax and abdomen. In the former, the pleuræ on the right side were extremely inflamed, and serum and pus were collected in their cavity. On the left side, the membrane was also inflamed, and serum and lymph, but no pus, were discovered. In the substance of the lungs, and especially the left, were tubercular masses of lymph and pus, numerous and varying in size. Similar deposits existed in abundance in the liver, and were seated, for the most part, immediately beneath the peritoneum investing the viscus. The intestines were generally sound, but the mucous membrane of the colon, near its valve, was injected and inflamed, though free from ulceration.

It is singular, indeed unaccountable, that the pleuræ and lungs should have been so *diseased* as they were, without having presented a symptom of *disorder*. The condition of the liver was productive of tenderness at the epigastrium, whilst the very highest degree of inflammation of a membrane like the pleura was totally unaccompanied with dyspnœa or pain! This is one of those things which may be "dreamt of," but will never, we fear, be comprehended "in our philosophy." It is probable that the rigors, malaise, and pyrexia, which set in on the 9th, sixteen days from the occurrence of the accident, were symptomatic of the formation of the depositions in the liver and the lungs, though cases not unfrequently occur, where no such particular indications are observed.

*Case 2.* Amputation above the Knee—Inflammation of the Pleura, and Abscess of the Lungs—Death on the 17th day.* A boy was admitted into St. George's Hospital, April the 9th, under the care of Mr. Brodie, with necrosis of the tibia and disease of the ankle. Tonic medicines were employed for a week or two, and amputation was performed above the knee upon the 24th, by the flap operation. A number of vessels required to be tied, and the boy was excessively irritable during the whole of the time.

For the first few days the appearance of the patient was unpromising; the tongue was brown; the pulse on the 25th 120, and on the succeeding days higher still, and indicative of great irritation; the surface hot; the countenance expressive of anxiety. The dressings were removed from the stump on the evening of the 28th, when it presented an unfavourable aspect. The tincture of benzoin was applied, and on the 30th, not only the stump but the symptoms altogether were improved, the tongue being cleaner, and the countenance less anxious.

*May* 1. The flaps of the stump have retracted, and the bone protrudes in consequence. Pulse 160, but not hard—tongue whitish—skin hot—less thirst. He is not quite so irritable as he was, and eats a little pudding with relish. On the 2d, his diet was changed to porter and roast meat, whilst a mixture of ten ounces of decoction of bark with ten grains of aromatic confection was given thrice daily.

*May 4th.* Circumstances occurred in the ward that exerted an unfavourable influence on the mind of the patient. He is anxious and irritable; pulse quick and bounding; tongue brown and red at the sides and tip; thirst; nausea; tenderness on pressure of the thigh or abdomen. The stump was not slow in partaking of the general disturbance; it was white, sloughy, and sensitive in the highest degree, and the bone protruded from its surface, for an inch or more. On the 6th he appeared to be moribund, the pulse being small, the countenance sunken, the mouth parched, the tongue dry and coated, and, to crown the whole, there was a harrassing diarrhœa.

℞. *Quinin. sulphat.* gr. jss.—*Acid. sulph. dil.* ♏iij.—*Aq. distil.* ℥vij.—*Tinct. Aurantii,* ℥j.—*Tinct. opii* gtt. iv.—*M. ter die.—Vin. rub. oss. quotidie.*

On the 7th and 8th, he appeared to be sinking, the disarrhœa persisted, the countenance was hippocratic. Eggs beat up in brandy, and red wine were administered largely, and chalk mixture directed to be taken till the purging should cease, which it did in the course of the day, though the mixture, by mistake, was carried on.

The symptoms persisted, for worse they could scarcely be; the integuments of the sacrum and hips became inflamed and excoriated, delirium set in, and he died on the 11th, having lingered surprisingly long.

*Dissection.* On raising the sternum, both the pleura costalis and pleura pulmonalis were found to be inflamed, and coated with a layer of apparently recent lymph. The adhesions were numerous and strong, most of them recent, others

* We alluded to this case at page 236 of the 17th number of the Journal, but with another object.

of older date. About an ounce and a half of sero-purulent fluid was discovered in the right side of the chest, but none in the left. The pleuræ were injected in parts, and every where more vascular than natural, particularly in spots corresponding to abscesses which had formed in the substance of the lungs. These abscesses were numerous, varied from the size of a hazel nut to that of a pea, and were scattered over the surface of both the lungs, immediately beneath the pleura pulmonalis. The heart was natural, the liver rather large but otherwise healthy, the gall-bladder distended, the mesenteric glands enlarged.

The stump had a sloughy appearance, and the bone was protruding for nearly two inches. Matter was collected in the cellular membrane between the origins of the adductor longus and magnus; the periosteum was detached, and in parts in a sloughy condition, and the purulent matter had insinuated itself to some extent between the periosteum and bones. The vessels of the limb were examined, but no deviation from health could be discovered in them.

The boy from the first was never doing well, but still there was a trifling amendment when the impression was made on his *morale*. This was on the 3rd, nine days from the operation, the time when the pulmonary affection is exceedingly apt to supervene. It may, therefore, be a question very difficult to solve, to which of the two we shall attribute the change, to the moral impression, or the latent and physical disease developing itself in the lungs. It is generally recommended to treat this affection (that is, when we can trace it) by active depletion; active in proportion to its insidiousness and rapidity of march. The rule may be a good one, but must have its exceptions notwithstanding, for in a case like the above, that was stamped from the beginning with the features of irritability and depression, we think there are few who would have ventured to deplete.

*Case* 3. A robust young man was sitting on the shafts of his cart, when he fell with his right leg under the wheel, and received a compound dislocation and fracture of the tarsal bones. He was brought to St. George's, and amputation performed below the knee, by Mr. Rose. This was on the 9th of June. A little hæmorrhage occurred after the operation, and in the evening of that day he was fidgetty and restless, with a flushed face, burning skin, and pulse above 100, and full. He was bled from the arm, and took thirty minims of the liquor opii sedativus, but he passed an indifferent night, and was disturbed in the early part by the starting of the stump.

On the 10th there were symptoms of strong arterial action, the pulse being 100, hard and full—the face high-coloured and bathed in perspiration—the tongue white and rough—the thirst rather urgent. *Saline with liq. ant. tart.* ℨss.—*mag. sulph.* ℨi. *sextis horis*.

On the 11th, the pulse, &c. had in some degree subsided, and the patient was better. This improvement, however, was, unhappily, a prelude to a worse state of things than before, for on the two succeeding days, the commencement of a change from excitement of the system to depression was perceptible to all. The pulse was as frequent but not quite so full, the tongue white and roughish, the thirst not so urgent, the morbid irritability excessive, and a quickness of manner the common forerunner of delirium. The dressings were removed from the stump upon the 12th, when its lips were discovered to be gaping, and its surface sloughy and white. Fresh strips of plaster and lint were applied in the first instance, but were changed on the 13th for the tincture of benzoin and cold water,* after which he was seized with a shivering, succeeded by heat and vertigo. He passed an extremely bad night, and suffered from a similar attack on the dressing of the stump next day. The pulse was now rather tremulous, but

* The tincture of benzoin is an excellent application to the surface of a stump that is disposed to slough. In one case which we witnessed, the stump had a most unfavourable aspect, and promised to slough most extensively, but the benzoin acted like a charm. The tincture should be poured into a gallipot, pieces of lint not merely dipped but soaked in it, and these should be placed upon the stump, which may also be *washed* with the benzoin. It is a favourite application of Mr. Brodie's in cases of this kind.

full notwithstanding—the skin was cooler but clammy to the touch—the tongue a little cleaner—the hurriedness of manner more marked.

15*th*. More composed; pulse not so frequent, but weak; countenance rather pinched and sallow; bowels open. Stump to be dressed with green digestive. *Quin. sulph. grs.* iii.—*acid. sulph. dil.* ♏. vi.—*tinct. zingib.* ʒi.—*infus. ros.* ℥iss. *tertia quâq. hor.*

In the afternoon he had a convulsive fit, succeeded by vomiting and rigor in the evening. The vomiting continued, though not with unabated severity, throughout the 16th, the surface getting cool, the pulse ranging from 110 to as high as 140, and the thigh being tender on pressure in the course of the femoral vessels. The night of that day was passed between dozing and delirium, and the next morning found him in articulo mortis. The secretion had ceased from the face of the stump, he was alternately sunk in a state of collapse, or raving under the influence of delirium, and thus he held out till the 18th, when he died.

*Sectio Cadaveris.* On opening the chest, considerable adhesions of the pleuræ were discovered, and the usual post-mortem appearances that indicate inflammation of the membrane. In the right lung, immediately beneath the pleura pulmonalis was discovered a yellowish mass, much harder than the neighbouring lung, which when cut into gave issue to particles of pus. There were many other little tumours of a similar description in both the lungs, but none of them so large as the preceding. The liver was large and two or three ecchymosed spots were discovered on its surface, though its structure was otherwise natural.

Besides these appearances in the thorax and abdomen, inflammation of the femoral vein had obtained from the stump to the profunda.

Here then are three cases of pulmonary abscess, two of them after operations, occurring at one hospital, in the course of two months, strong presumptive evidence, or rather proof positive, that the affection is far from uncommon. Mr. Rose in his paper observes, that in all the cases which he had seen, "these abscesses took place at some period between the end of the second and that of the fifth week after the accident which gave rise to them." There is a degree of obscurity in the passage, as we cannot determine to which Mr. Rose is alluding—the *formation* of the abscess, or the period of its fatal *termination*. If the former is intended, its accuracy as a general position is invalidated by two of the cases we have recorded, in one of which the unfavourable symptoms commenced on the tenth day, and in the other the patient was *dead* on the ninth. Mr. Rose likewise says that the abscesses are most apt to form, when the parts in which the injury took place are in a state of suppuration, and farther, that "a favourable change has frequently taken place in the wound, before the symptoms of the internal abscess have begun to manifest themselves."

As general rules, the above, without doubt, are correct, but then they are not corroborated by the cases we referred to before, in both of which the stump was unhealthy and sloughy, instead of being in a state of suppuration. Whatever is the state of the stump or the wound *before* the affection of the lungs becomes established, the secretion is afterwards affected, in some cases entirely arrested.

We hope to be excused for extending this paper to the length we have done, but the interesting nature of the cases recorded, and the general importance of the subject will we trust be sufficient apology.

---

### 46. EPIDEMIC ERYSIPELAS.

### [Hôpital de la Pitié.]

At page 284 of No. XVII. we alluded to the prevalence of erysipelas at La Pitié, and related some cases in which blisters had been used. The epidemic continued for nearly a month, and has furnished the subject of a second report. As it happens rather oddly in this new batch of cases, that not a solitary blister was employed, but the treatment adopted was leeching and bleeding, we revert to the subject without any fear of repetition, but as much of variety as a theme like erysipelas can afford.

*Case* 1. A young man of a good constitution and sanguineous temperament had complained of sore throat for some days, when he was seized on a sudden with vertigo, and fell to the ground in a fainting fit. Twenty-five leeches were applied to the anus, a poultice to the neck, and sinapisms to the feet with pediluvia. This was on the first of May, and on the second, erysipelas appeared upon the face.

On the 3rd, he was admitted into hospital, the erysipelas being of a bright red colour, the pulse nearly natural, the tongue white and reddish at its edges, the skin hot and dry, cephalalgia. There was neither nausea nor vomiting, but there was soreness of throat, and the velum palati and bag of the pharynx were redder than natural. He was bled from the arm in the morning, and in the evening five and twenty leeches were applied to the neck. The erysipelas spread over the face, and the bleeding was repeated on the 4th and 5th. On the 6th, the neck became affected, but the colour of the inflammation was paler; the skin was intensely hot; the tongue dryer; no pain of abdomen nor vomiting. *Leeches to the anus.* On the 9th, the patient was light-headed, and this increased on the 11th, to such furious delirium that the strait-waistcoat was put in requisition. The erysipelas had deserted the face and was principally seated on the chest, while a separate blush had appeared around the lancet-wound made in the arm. The stools were passed in bed, the teeth incrusted, the expression haggard. Antispasmodics and lavements were employed, and gradually the patient revived from the prostration and collapse in which he lay, the tongue got moist, he complained of weakness, and asked for food. On the 18th, the erysipelas had completely disappeared, and the patient was convalescent, but it returned on the 10th of June, "travelling" rapidly from part to part. Leeches were placed upon the epigastrium, and on the 26th, when the report was published, the patient was in a very debilitated state.

The treatment of M. Lisfranc appears to have been judicious, which is more than we can say for the remarks of the reporter. It may be, however, that the bleedings and leechings might, perhaps, have been ordered more sparingly, and salines, with diaphoretics, exhibited instead.

*Case* 2. This was likewise a young man of robust and sanguineous habit, who was attacked with erysipelas of the face, and received into La Pitié on account of the affection. Three bleedings were employed, with emollient drinks, and "sinapised" pediluvia. On the eighth day from its first invasion, the erysipelas had completely disappeared.

The above were both patients of strong constitutions, who were seized with erysipelas at home, and entered the hospital *for* the affection. In cases of that kind, depletion can be borne, and is often imperiously required, whilst in those who have long been the tenants of a hospital ward, pulled down by disease and the doctor, the erysipelas, when it comes, is marked, for the most part, with the features of debility, and is not to be "put out" by a squadron of leeches, or the lancet.

The epidemic which prevailed at La Pitié, where the surgical wards are on the ground, and proverbially unwholesome, appears to have been marked, as epidemics invariably are, with particular characters. A case or two may serve as the best illustration.

*Case* 3. A young man of a lymphatic habit of body, who entered for ophthalmia, after some general bleedings, had a seton applied in the nucha: in the course of a week, erysipelas appeared on the back of the neck; the skin was hot and dry; the tongue was invested with a yellowish fur, and red at the edges; the epigastrium sensible to pressure, if applied with any force. *Gum water—thirty leeches to the epigastrium.* The erysipelas abandoned the neck, and spread upon the back; then attacked the face, and, finally, expended its ire on the nose, where it left a few mementos in the shape of vesications.

*Case* 4. A young woman of scrofulous habit, with enlarged cervical glands, had also a seton applied in the nucha, for ophthalmia, and was seized with erysipelas, which spread over the shoulders, and attacked the face, where it continued for several days. The skin was hot and dry; the tongue thickened, white in its centre, and red at the border; no vomiting, nor nausea. Leeches were applied to the epigastrium, and after their application, tenderness of the part was observed upon pressure. Frequent cough came on, with anxiety, difficult respiration, frothy expectoration. *râle sibilant* at the apex of the right lung, *râle sonore* mixed up with the *râle sibilant* at its basis, and *râle sonore* over the whole of the left lung. A fresh relay of leeches was put on the epigastrium, when the symptoms of bronchitis subsided, and the erysipelas likewise disappeared.

From what we have detailed, it will be evident that a degree, more or less perceptible, of gastric derangement, or "gastricity," as M. Lisfranc has termed it, was a feature in the hospital epidemic. The tenderness of the epigastrium and state of the tongue were warranty enough for the use of the leeches, though scouted in toto by the Parisian reporter, who quarrels with the surgeon for using any remedies at all!

Thus it is ever with the million! Semper in extremis is their motto—the medicina perturbatrix on this side the Channel—the medicina expectans on that!

---

### 47. AMAUROSIS—"AMAUROTIC CAT'S EYE OF BEER."

[St. Bartholomew's Hospital.]

The subject of the above very singular heading was a delicate barber of 23, who was under the care of Mr. Lawrence.

About 8 years previous to admission, the left eye began to be affected, and the right in the last nine months. The vision of the former was so far destroyed, that the patient could barely distinguish light from darkness, but with the latter he could read large print. The anterior chamber in each was unusually small, the iris dark brown and transparent, the pupil half contracted, sluggish, and dull. On dilating the pupil of the left eye, a grey or dull yellow concave opacity was observed, with blood-vessels ramifying on it. In the right, the opacity was limited, and the blood-vessels not to be seen. Mr. Lawrence observed that the amaurosis was dependent on some organic disease, but it was difficult to determine whether it consisted in a change in the retina, or a want of the pigmentum nigrum.

The patient was cupped in the back of the neck, and five grains of blue pill administered twice in the day. Nothing further was done for him, and the patient was dismissed at the end of a month, no better than when he was admitted.

We shall here introduce two cases of amaurosis, which were treated at La Pitié with greater success than the above.

### AMAUROSIS SUCCESSFULLY TREATED BY THE AMMONIACAL OINTMENT.

M. Lisfranc, at the hospital in question, has been giving a trial to Gondret's ammoniacal ointment, and it seems that, in cases arising from an affection of the fifth pair of nerves, the remedy has proved of considerable service.

The ointment is in general applied upon the forehead, as the frontal ramifications of the fifth pair are distributed thereon after traversing the orbit. In the following cases the ointment was employed, and relieved the amaurosis.

*Case* 1. A soldier of robust constitution, who had formerly campaigned in hot countries, and been exposed, in bivouacking, to the cold night air, found his vision grow feeble in the course of 1824. In the following year, he was unable to see his way about the streets, and was directed to have a seton in the neck, some leeches to the temples, and ordered some pills, composition unknown. His sight was improved by the measures employed, and he was able to distinguish various objects pretty well. The amendment, however, was temporary only, and, on the 10th of last January he entered La Pitié. He was bled from the arm, and ordered an emetic, but without effect; he could barely distinguish light from darkness—the pupils were dilated, but acted feebly, and the left was more sluggish than the right. On being questioned respecting the history of the disease, he replied that, in the first instance, he could see rather better towards evening or twilight than in mid-day; he could also distinguish more clearly any object above him, than one which was below, or on a level with the eyes.

On the 24th of January, the head being shaved, the ammoniacal ointment was applied upon the forehead, and its employment continued till the 17th of February. Pustules were formed, which ulcerated deeply, and the case was proceeding in a favourable manner, when a severe erysipelas seized upon the head, and required the most active depletion. The patient was bled seven times in the course of as many days, and sixty leeches were applied besides. The erysipelas disappeared, and the powers of vision were exceedingly improved; in the right eye, indeed, they were entirely restored. The left, which was always more affected than the other, was scarcely, if at all improved.

*Case* 2. A tailor, ætatis 39, had laboured under amaurosis for two years past, and was admitted into hospital, incapable of seeing at all. The pupils were dilated, and contracted but feebly on the approach of light—the patient had the peculiar undefinable aspect which invariably marks the amaurotic—the globe of the eye was continually rolling in the orbit, and was, in general, rotated upwards; and, lastly, the power of directing the organ, particularly sideways, was already beginning to be lost. The disease had come gradually on, and the patient attributed its origin to his business requiring him to work much by candle-light.

The ammoniacal ointment was applied upon the forehead and crown of the head, and its use was continued for upwards of a fortnight. Abscesses were formed, and converted into healthy ulcerations, which furnished a copious discharge. The good effects of the *issue* were quickly apparent, for, first, the patient was enabled to distinguish the day-light, then regained the power of discerning the objects around him, and finally, at the expiration of a couple of months, his sight was as perfect as before the amaurosis commenced.

The reporter observes that, in order to prove beneficial, the application of the ointment should be always persevered in, till more than superficial ulcerations are produced. In both of the cases recorded above, the disease was very far from being recent, and required the treatment to be continued, in one case, for four months, and in the other for nearly as long.

It is curious that the soldier "could distinguish more clearly any object above him, than one which was below, or on a level with the eyes." It was imagined by Richter, in similar cases, that a point of the retina still continues sensible to the impression of light, and he cited the case of a patient, who was otherwise blind, but retained the extraordinary power of perceiving an object above the ala nasi. His vision was limited, but so perfect, notwithstanding, at this particular point, that he saw, at a considerable distance, the arrow on the top of a steeple though it always required much adjustment and balancing beforehand. The dilatation of the pupils is a frequent occurrence, and Mr. Ware has observed that the above has been commonly the case when electricity proved beneficial. The disease altogether is attended with such curious and frequently anomalous characters, and its treatment is so often unsuccessful, that any contribution to the scanty stock of *remedies* (not drugs) must prove an acquisition of value.

### PUNCTURE OF THE RECTUM.

It appears that a boy, having been knocked down by a horse and cart, received a cut on the head, and was carried in a state of insensibility to St. Bartholomew's Hospital. Calomel and jalap were ordered, and on the following day he became sensible; but as his bowels had not acted, the calomel and jalap were repeated, and were followed in the middle of the day by an enema. The boy appeared to suffer greatly the remainder of the day, and died at eleven that night. On dissection, there was found a "slight fracture of the base of the cranium, with effusion of blood beneath the dura mater." On opening the abdomen, a quantity of light brown fluid was discovered in the cavity of the peritoneum, "which exhibited marks of inflammation;" and on prosecuting the examination, a "hole" was observed in the anterior part of the rectum. The "light brown" fluid, we are told, eventually proved to be oil, and the "hole" to have been made by the glyster-pipe, which was of a "most unwarrantable length, namely, seven or eight inches."

It may seem altogether incredible, that a case like the above could be converted into a handle for the abuse of "our charitable institutions," and the medical officers attached to them. So, however, it is; and amongst other good things, we are told that cases similar to this in

*atrocity* are constantly occurring at our public hospitals. A person who had any pretension to a knowledge of medical matters would pause awhile, perhaps, before he attributed the death of the boy to the effusion of oil in the cavity of the peritoneum. The enema was administered in the middle of the day, and the patient was dead by eleven at night, a very rapid termination indeed of peritoneal inflammation! The bile, we should imagine, is rather more acrid than oil, and, in the celebrated cases of wound of the gall-bladder and effusion of its contents into the cavity of the peritoneum, recorded by Dr. Steward and Sabatier, the patients survived several days, one of them as much as a week. A boy was admitted in the Spring into St. George's Hospital, who had been shot in the belly with a fowling piece. The colon was wounded, and the fæces extravasated in abundance, but he lived, notwithstanding, for three days at least. It is utterly repugnant to the dictates of experience, or even common sense, to imagine that, in ten or eleven short hours, the inflammation of the peritoneum should have run its course in a healthy boy.

Granting, however, that the patient *did* die of the enema, still the nurse and not the surgeon was at fault. Do these medical reformers intend that the glysters should be under the immediate direction of the surgeons or physicians, or wish these to be responsible for all and every of "the negligences and ignorances" of their subalterns? The doctrine, at any rate, is recent, for we remember not very long ago, when a celebrated surgeon, the Coryphæus of his party, lost a patient by bleeding from the cuts which he had made in erysipelas, absolution was pronounced on the gentleman himself, and the blame very quietly laid on the back of the dresser. The change, we suppose, is attributable to the stride which has been taken by the "march of intellect," and nothing can be wondered at in an age of improvement like this! The accident occurred under the care of Mr. Vincent; but Mr. Lawrence, of course, never thinks of confiding so *delicate* an operation to any other hands than his own!

---

### 48. Rupture of the Ileum.

[Guy's Hospital.]

An Irish labourer had the handle of a hand-cart driven with violence against his abdomen, on the 30th of June. He vomited, and in the course of an hour was seen by a surgeon, who bled him and gave him some pills. When taken to the hospital he had the usual symptoms of ruptured intestine, but so slight as to occasion a doubt as to the actual nature of the accident. Hot water was applied to the feet and the belly fomented, but next day the countenance was sunken; pulse 90, hard, sharp, and jerky; vomiting of a greenish fluid; thirst; pain and tenderness of abdomen; tongue furred and dry. Purgatives were given and leeches applied; but at 5, P.M. of the 2d, the patient expired.

On dissection, the marks of peritoneal inflammation were extremely intense; but, on tearing asunder the masses of lymph, the upper part of the ileum was found to contain two apertures with ragged edges, each of them about two-thirds of an inch square. The mucous membrane was not everted at the apertures, both of which were situated at the mesenteric side of the intestine, close to the point where the latter made a turn upon itself. One of the rents at the apex of the angle was perfectly open, the other, about half an inch lower, had opposite to it, on the other convolution of the intestine, a "large piece of lymph," which appeared to have fitted in the aperture, and plugged it up. There was an immense extravasation of fæces and serum into the cavity of the abdomen.—*Gazette.*

This case will bear upon the one which occurred at Bartholomew's Hospital; for here there was effusion enough in the cavity of the peritoneum, and yet the individual survived for upwards of 48 hours, instead of being carried off in ten! The reporter makes some sensible remarks upon the dangers of purgation in cases of ruptured intestine, a measure which inevitably tends to destroy what adhesions may form, or prevent their formation altogether. The gentleman appears to be rather more sanguine in his expectations than many, and entertains that HOPE which is said to come to all, even in so fatal an accident as this. He concludes some remarks by observing that rupture of the intestine is not necessarily fatal, and adduces a case in the following number of the Journal we quote from, as affording a satisfactory confirmation of the truth of his opinion. The case is as follows.

A lad of a delicate habit was crushed between a cart and some palings at six in the evening of the 9th of July. He fell to the ground, the surface was blanched, and he vomited immediately afterwards. Bottles of hot water were applied to the feet, and when admitted into hospital the features were anxious and shrunk, breathing but little affected, abdomen, the seat of pain, swollen, and tender upon pressure, pulse quick, small and weak. Being incapable of making water, the catheter was used, and warm fomentations applied to the abdomen. On the morning of the 10th, re-action had occurred, the face being coloured—the surface warm—pulse 130, small and weak—tongue a little furred. The abdomen was swollen, painful, and tender, especially on the left side, and a circular spot existed in the right hypochondrium, which was buffy, as if air or fluid were within it.—*Twenty leeches to abdomen—calomel and opium.*

In the evening the pulse was 150, small and sharp—vomiting at intervals, with some hiccup—extremities warm—countenance anxious—abdomen not very painful nor tender, but tense and swollen—*Calomel and opium repeated—four ounces of blood from the arm*—12 *leeches to left side.* He passed an indifferent night, and on the 11th the pulse was quick, small, and rather sharp, frequent vomiting. More leeches were put on, and the infusion menthæ comp. administered to allay the vomiting, which it did when two ounces had been taken. In the afternoon he was in more pain, had vomited again, and was troubled with a little hiccup. Leeches were again applied, and there being no stool, Mr. Callaway directed the nurse to throw up an enema, and especially *to use no force.*

In half an hour a solid healthy motion was the consequence, but the abdomen still continued tense—pulse 130, small and rather hard. *Poultice to the abdomen. Rep. pil.* 12*th.* Has been restless and a little delirious—at present free from pain—pulse 120, and sharper—tongue brownish—abdomen less tense and swollen, (he had had a fluid motion,) except on the left side little tenderness. *V. S. ad* ℥vi.—*Hirud.* xij.—*Infus. menth.*—*Cal. c. op.* There was no motion that day, and at night another enema (with the same *precautions*) was thrown up. 13*th.* Better; but no stool yet. *Leeches, enema, and calomel and opium repeated.* He passed a quiet night, and on the 14th had several motions, indeed some purging supervened, but was stopped by the mistura cretæ. The patient soon recovered.

The reporter observes, that the early and remarkable swelling which occurred, can only be accounted for by supposing extravasation into the abdominal cavity. It is easier in cases of this kind to say what has *not* taken place than what *has*, and, for our own parts, we do not believe that the ileum was ruptured or its contents effused. The most prominent, we might almost say pathognomonic indications of ruptured intestine, are the extraordinary prostration and collapse which supervene—the coldness, paleness, and depression—the feebleness of pulse—and, lastly, the almost universal fatality of the accident. In the case which is the subject of report, the depression had, in twelve or fourteen hours, passed away, and the symptoms altogether had not that intensity of character which they speedily assume in cases of effusion. That some degree of peritonitis was the consequence of the injury, (whatever that injury was,) is evident from the abdominal tension and pain, but the reader of the case cannot fail to be struck with the obstinate costiveness at first, and the immediate relief which was obtained, when the bowels were effectually opened. If we couple with this the *gradual* accession of the swelling of the abdomen, it is not too much to *suspect* that a purgative or enema administered earlier might have saved a few dozen of leeches to the belly, and some hours' uneasiness to the patient and surgeon.

---

### 49. HYDATID CYST IN THE LIVER, OPENING INTO THE RIGHT PLEURA.

[La Charité.]

*Case* 1. A female servant, 42 years of age, was admitted into La Charité on the 28th of March, and placed under the care of M. Fouquier. The colour of the skin was icteritious—the stomach was disordered—there was a dull pain in the right hypochondrium, and the liver could be felt beneath the ribs, whilst its left lobe especially was greatly enlarged, and reached to the level of the umbilicus; its surface was regular, but painful on pressure.

The history of the case was as follows:—Three years before she was admitted, she began to suffer pain in the right hypochondrium, accompanied with symptoms of indigestion. The pain, &c. subsided in the course of a little while, but reappeared at intervals afterwards. In March, 1827, the pains were severe, she lost her appetite, was attacked with fever, and the surface for the first time assumed an icteritious hue. Appropriate remedies were employed, and the symptoms disappeared, but left behind them debility, disorder of digestion, a sallow complexion, and white-coloured stools. Three weeks before she applied to the hospital, her sufferings were aggravated, the febrile disturbance was great, and she was troubled with vomiting of a bitter and yellow-coloured liquid.

The history of the malady and symptoms that were present left no doubt whatever that the case was hepatitis, chronic in the commencement, but degenerated into acute. The treatment consisted in bleedings from the arm, emollient ptisans, and antiplogistic diet. The patient was relieved, but complained, on the 4th of April, of bilious vomiting, pain in epigastrio, constipation, and sleeplessness. *Leeches to the anus—emollient glysters.* The vomiting of bile disappeared, but still there were nausea, dry coated tongue, quick and sharp pulse, hot skin, and scanty urine. The leeches to the anus were repeated, but on the 10th the vomiting returned with unabated severity, the emaciation was rapid, the countenance anxious, decubitus on the left side, attended with pain.

*April 12th.* The patient complains of excruciating pain in the right side of the chest. The respiration is hurried and oppressed, the cough very troublesome, and accompanied with expectoration, consisting of a mixture of reddish froth and thick green matter, which falls to the bottom of the pot. The respiratory murmur was distinct on the right side throughout; as much so, indeed, as on the left. The vomitings ceased, but the patient continued to expectorate greatly; and on the 16th presented the following stethoscopic indications. The sound, on percussion, at the base of the right side of the thorax, is bad; the respiratory murmur gone, but the sound at the upper part is clearer than on the opposite side. During inspiration, coughing, or, when the patient speaks, a well-marked bruit amphorique is heard along the base of the scapula, the passage of air into the large ramifications of the bronchi is still distinguished; whilst reclined upon the back, the right side gave a tympanitic sound—the metallic tinkling was distinct behind.

From this period the patient continued to decline, the cough and expectoration were frequent, and the latter, a day or two before death, became more fluid and assumed a yellow tint; the respiration became so difficult, that the patient was obliged to be constantly supported in bed, and on the 25th of April she expired.

*Sectio Cadaveris.* The enlargement in the situation of the liver had entirely disappeared. On opening the abdomen, the right lobe of the liver was found to be changed into an extensive cavity, the walls of which were half fibrous, half cartilaginous, with a very thin layer of the proper parenchyma externally. This cavity was filled with a yellowish fluid, of a fetid odour, and a quantity of hydatid cysts, varying in size from that of a marble up to a large hen's egg. At the spot where the liver and diaphragm are in contact, without the intervention of the peritoneum, was an opening that would readily admit the fore-finger, between the cavity in the liver and the right lung. This fistulous opening in the lung led partly into several ramifications of the bronchi, and partly, by another distinct aperture on the outside of the lung, into the cavity of the pleura. The lung was considerably reduced in size, flattened against the mediastinum, and extensively adherent to the diaphragm; it was almost deprived of blood and crepitated no longer. The pleura was inflamed, invested with a layer of lymph, and contained in its cavity a quantity of the same kind of fluid as was found in the liver, and a number of hydatids.

The left lobe of the liver was enlarged and its substance preternaturally soft; the stomach and duodenum presented marks of inflammation. In the left ovarium was a cyst, about the size of an egg, the walls of which were dense, and contained a liquid, in colour and consistence very similar to chocolate. The right ovarium also presented a cyst, but its walls were transparent, and the fluid was limpid and colourless. The rectum, about three inches from the anus, was so compressed by these ovarian tumours, that its calibre was reduced to the space of half an inch.

The reporter remarks that the formation of hydatids in the liver cannot be considered a very rare affection, though the origin of the disease is involved in the deepest obscurity. When, however, the affection is advanced, the gentleman conceives that a careful and anxious examination will frequently detect its existence. The diagnosis is important, as the disease, though too frequently fatal, occasionally admits of relief from art, and even has been cured when it opened spontaneously across the abdominal muscles. In the celebrated case where professor Recamier hastened the opening of the sac by means of the potassa fusa, the *issue* was successful. In a case which occurred at St. George's Hospital, the cyst was either opened by the surgeon, or burst of itself, (we are not certain which) and a cupping glass was applied with the immediate effect of extracting an extraordinary quantity of hydatids. The patient died.

Had the existence of the cavity in the liver and its contents been inspected when the subject of report was admitted into La Charité, it would not have been a case, notwithstanding, for the interference of art. The total destruction of the right lobe of the liver, and the general condition of the patient would have rendered an opening in the walls of the abdomen peculiarly hazardous, and probably speedily fatal. The communication of the cyst with the bronchi and pleura was indicated by the nature of the expectoration, and fully established by the evidence of auscultation and the stethoscope.

*Case* 2. *Conversion of the Right Lobe of the Liver into a vast Hydatid Cyst—fatal Peritonitis.* A middle aged woman ceased menstruating at 43, and shortly afterwards began to be affected with deep-seated pains in the right hypochondrium. The digestion was good, the pains only troubled her at intervals, and she paid very little attention to her case. In the course of last August, the pains became severe, the belly grew tumid, and the surface acquired a copperish tinge; the appetite, however, continued, and the digestion was unaffected. In October, the patient had diarrhœa for a fortnight, and from that time began to emaciate, and complain of loss of appetite. The diarrhœa returned several times, and in December, 1827, she entered La Charité, presenting the following symptoms; commencing marasmus—yellow tint of skin—tumefaction of the abdomen, and some degree of effusion into the cavity of the abdomen. The liver on the right side was felt below the ribs, and its left lobe extended as far as the umbilicus. The surface of the organ was smooth, and the pain was not increased on pressure; there was a very uncomfortable feeling of weight in the region of the epigastrium—appetite tolerable—digestion pretty good, when the quantity of aliment was small—pulse rather feverish—skin dry. Gum-water was ordered with nitrate of potass and frictions of hydriodate of potash, under the influence of which the dropsical effusion diminished. Leeches were applied to the anus, but on the 18th of January, the abdomen was painful and tense—the tongue dry and brown—the patient became delirious and on the night of the 19th she died.

*Sectio Cadaveris.* On opening the abdomen, two pints of serum were discovered in its cavity, and the peritoneum in several places shewed traces of recent inflammation. The liver had acquired an extraordinary volume, and pushed up the diaphragm to the level of the cartilage of the fourth rib. Its convex surface was smooth and covered with layers of lymph. The right lobe was converted into one enormous cavity, with thick and fibrous walls, which was filled up with white, inodorous pus, containing in its centre hydatids of different dimensions and perfection. The parenchyma was totally destroyed on the concave surface of the lobe, on the convex a layer existed, from two to four lines in diameter. The left lobe was double its natural size, but its structure was sound, and the gall-bladder was filled with bile, the composition of which was apparently sound. The stomach and duodenum were sound—the large intestines a little inflamed. The lungs were unaffected, the heart was small and flabby and contained some loose coagulum.

It is curious, though far from uncommon, that, notwithstanding the existence of organic disease in the liver, the appetite should have continued unaffected, and the operations of digestion, to all appearance, undisturbed, to a very late period of the patient's existence. It is probable, as the intelligent reporter suggests, that the cavity in the liver contained in the first instance, only hydatids, and that the purulent matter was formed at the time when the marked aggravation took place in the symptoms. From the healthy condition of the left lobe of the liver, the above would have afforded to the operator a much greater promise of success than the case at the head of the report. In the present state of knowledge, with regard to organic disease of the liver, we fear there are few who would venture from the catalogue of symptoms presented by this patient to plunge a trochar in the hypochondriac region on the speculation of arriving at an abscess or cyst of hydatids! Our only instructor is experience, a tardy but certain preceptor, and by means of the PRESS, the experience of one, if rightly recorded and faithfully studied, becomes the experience of many. With this object in view we have minutely reported the features of these interesting cases, and hope they may be useful in aiding a doubtful diagnosis.

---

### 50. AMPUTATION AT THE HIP-JOINT.

[Hôpital de Montpellier.]

There was a time, 'tis *not* sixty years since, when amputation of the hip-joint was considered by many as the *ultima thule* of temerity, and even inhumanity. Mr. Pott, in speaking of the operation, observed, "that it is not impracticable (although it be a dreadful one) I very well know: I cannot say that I have ever done it, but I have seen it done, and am now very sure I shall never do it, unless it be on a dead body." It is to the surgeons of the army and navy that science is indebted for its establishment, for although it had been treated on, and even practised, previous to the late Continental war, yet then, and not till then, the experiments were made on such a scale, in our own and the enemy's ranks, as to entitle it to be considered a standard, though still very formidable operation.

The veteran Delpech has performed it with success at Montpellier.

*Case* 1. In the month of June, 1823, a young man applied at the hospital, with hardness and tumefaction of the right thigh and a number of sinuses leading in different directions to loose and dead portions of bone. Abscesses had formed and been discharged at different times, and left very great induration of the soft parts in their train. It was clear that there existed an extensive necrosis of the thigh, but the bone was affected too high to admit of amputation of the limb, and the constitution beginning to suffer from the local irritation, it was determined to perform amputation at the hip-joint.

As a preliminary to the operation, the femoral artery was tied. Instead of making two flaps, as had hitherto been done, M. Delpech had resolved to cut the inner one larger and longer than usual, and turn it over the acetabulum, so as in some measure to fill up the cavity by the great mass of muscle, and prevent, as far as possible, the access of air. This intention, however, he was unable to execute as completely as he wished. The inner flap was cut of a suitable size, by turning the knife from within backwards, so as to comprehend in its sweep the greater part of the muscles on the back of the thigh. The limb was, in the next place, forcibly abducted, and the inner half of the capsule divided with the knife, but at this stage of the operation, the bleeding was so free from one of the collateral vessels, that a ligature was required, though the femoral artery had been previously tied. The thigh being drawn inwards, the knife was "set on" somewhat lower than the middle of the glutæi, and carried obliquely down to the capsule, so as to form an outer flap, but of trifling dimensions. The remainder of the capsule was divided, the head of the bone disarticulated, and the limb removed. Two vessels only were secured at the second stage. The efforts of the surgeon were now directed to the closure of the wound as quickly as possible, its surface being such, that no one, in M. Delpech's opinion, could survive its suppuration. The infiltrated and consolidated state of the parts which composed the inner flap presented a considerable obstacle to its being drawn backwards, but at length it was effected by means of perseverance. The interrupted suture was passed through the integument, (and integument only) and the flaps brought together, except at the outer part of the acetabulum, where they could not be got to unite. A compress of lint and a bandage completed the dressings.

The day after the operation, an abundant discharge of serous fluid took place, and completely soaked through the dressings. In the course of some days, however, it gradually ceased—the two flaps united by the first intention, except at the outside of the acetabulum, where a healthy suppuration took place, and, at the end of a month, the patient had completely recovered, with the exception of a trifling discharge.

In the course of last April, the man was presented by M. Delpech to the Royal Academy of Medicine. He appeared to be perfectly cured, and could walk with facility on his wooden leg.

*Case* 2. The issue of this was unfortunate, the patient refusing to submit to an operation till the favourable period was past.

The affection that induced M. Delpech to have recourse to so formidable a measure, was an old and ununited fracture at the upper part of the femur. Prior to the performance of the operation the patient was attacked with acute peritoneal inflammation, and though the operation itself was successful, the abdominal inflammation assumed a chronic character, and, unluckily, proved fatal at the end of eight months. There was none of the consolidation and thickening of the textures of the limb, which were present in the former instance, and, therefore, the surgeon was able to execute fully the plan he had proposed. The flap on the inside was cut longer than before, and the outer one was really no flap at all, the incision being curved, and nearly on a level with the crest of the ilium. The skin from the inside covered the glutæi muscles, the fleshy part of which was saved in considerable quantity, the adaptation of the parts on the face of the stump was complete, and, on the 20th day, the wound was entirely healed, without the most trifling suppuration! On dissection of the stump, after the death of the patient, the acetabulum was found to be filled by the soft parts that constituted the centre of the internal flap, the connexion between them being formed by the medium of a firm and condensed cellular tissue.

M. Delpech recommends the prior securing of the femoral artery, on account of the size of the vessel, and the "uncertainty of the measures adopted for the pupose of compression." His reason for not employing a flap on the outside as well as the in, is the little disposition to unite with the acetabulum which exists in the tendons of the glutæi, and the extreme liability to suppuration. It is important, in the opinion of the operator, to bury the acetabulum in the flesh of which the inner, or, more properly, the *only* flap is composed, which may easily be cut both as thick and as long as is desired, on account of the number of muscles that exist on the inside of the thigh. The comparative result of the first case, where a double flap was made, and the second, where there was only the one, is adduced as a proof of the evident superiority of the latter, with the view of preventing suppuration.

We leave the proposal to the consideration of such of the profession as are immediately interested in the question. To us it appears an important suggestion, and its coming from a practical man, who has operated twice with success, must give it some weight with his practical brethren. Whilst we join in the meed of approbation which was reaped by M. Delpech when he developed his sentiments, and shewed the living record of his skill, at the Academy of Medicine, we doubt, at the same time, whether the nature of one of the cases, at any rate, was such as to *demand* amputation at the hip-joint. The one we allude to was an instance of necrosis of the femur, and we see no good reason for not having amputated high in the femur, and removed the sequestra, if such had been found to exist. The medullary cavity of the femur does not extend as high as the trochanters, and the neck of the bone is but seldom involved in the disease. On this account we are disposed to agree with Mr. Bell, and consider amputation of the hip-joint as generally uncalled for in cases of necrosis. If the neck and the head of the bone are diseased, the soft parts, we imagine, would be implicated too far to allow of much prospect of success.

In the case where the operation was performed with success by Mr. Orton, the thigh-bone was diseased in its whole length, and abscesses extended to the dorsum of the ilium; the result was more fortunate than the surgeon might expect.

The particulars of M. Delpech's second case, are so briefly detailed, that it is difficult to form any estimate on the necessity of resorting to the operation. Be this as it may, the operation itself must be considered successful, the patient surviving eight months, and dying at last from a different cause. These cases then, added to the number of those that are already upon record, make five that have recovered out of twenty-two; a proportion which is calculated to make the hip-joint operation by no means so desperate as it is frequently considered.

The Frenchman on whom Mr. Guthrie operated after the battle of Waterloo, we find, by the Journals, is still at the Hôpital des Invalides, and frequently referred to by Larrey!

---

### 51. HORÆ LACONICÆ.

### No 2.

### NEW THEORY OF THE CIRCULATION.

HARVEY overcame many of the difficulties attendant on the circulation of the blood, and his doctrine, in all important particulars, stands established and confirmed by TIME. Some difficulties still exist, as to the return of blood by the veins:—and our intelligent countryman, BARRY, has done much to lessen these difficulties. It remained, however, for a personage who stiles himself—"FERD. LAU, *London*," to achieve what Harvey and Barry failed to do; and to shew that the GLOTTIS is the auxiliary agent to the HEART in the venous circulation! The theory is so beautiful, and at the same time so LACONIC, that it must have a place in the HORÆ LACONICÆ.

"Professor Mende (of Gottingen) published a little treatise on this subject, in which he says that the glottis is closed, and opened, alternately, by two bodies resembling the lips, in the act of shutting of which there seems to be some degree of power; and to make the closure still more complete, the epiglottis lays down on the glottis; likewise, as if by some muscular action, the closure takes place between each inspiration, and expiration, and lasts longest after the expiration.

"It appears, then, that during the closure of the glottis, the communication between the air-canals of the lungs, and the external atmosphere, is entirely suspended; or that the closure is air-tight, which is also particularly favoured by the labial structure of the parts. Now it is my object to show, what the effect of this closure of the glottis must be on the circulation of the blood. We know that a part of the air is absorbed by the blood in the lungs; and are we, therefore, not entitled to conclude, that a vacuum is formed in the bronchi during the closure, and must not such a vacuum evidently have a powerful effect on the fluid blood, which thus, by atmospheric pressure, is compelled towards the vacuum? The pressure of the atmosphere chiefly acts upon the abdomen; the moveable contents of which, and the blood of the large veins, are pressed into the pectoral cavity, until the glottis is opened again, when the equilibrium is restored. This reasoning, I think, is founded on such indisputable principles, that there will be no difficulty in conceiving it."*

Whatever difficulty there may be in *conceiving* this *reasoning*, there can be no doubt that it is now happily *brought forth*—and a precious *conception* it is! When Dr. Barry sees this new AUXILIARY come into his ranks, he will probably exclaim—

Non tali *auxilio*, nec defensoribus istis,
SANGUIS eget.

* LANCET, No. 255, p. 502.

52. Case of Rupture of the Stomach produced by Vomiting; with some Observations. By J. N. Weekes, Esq. House-Surgeon to Bartholomew's Hospital.

[Med.-Chir. Trans. Vol. XIV.]

Rupture of the stomach, independent of external violence or internal disease, is so extremely rare an occurrence, that we question whether the present case be an exception to general experience. We shall state the particulars of the case, however, before we offer our reasons for this opinion.

George Andover, aged 34, had been liable, for about two years, to paroxysms of pain in the stomach, generally ending in vomiting, and returning at uncertain intervals. Some of these intervals amounted to several weeks, during which the patient enjoyed tolerable health. About Christmas, 1827, he vomited "*a large quantity of blood*," which rendered him so feeble that he was confined to bed for five weeks. From that period his health was much impaired, "and the attacks of pain, followed by vomiting, were more frequent." On the 13th April, 1828, he entered Bartholomew's Hospital, suffering great pain, extending from the epigastric region over the whole abdomen, and accompanied by nausea, but without tenderness on pressure, or tension. Pulse frequent, tongue clean. He attributed these symptoms to a draught of shrub and water. Next day the pain had subsided—no vomiting, but complained of nausea—abdomen distended—frequent eructations—pulse weak. At 11 o'clock, p.m. he had a sudden attack of severe pain at the pit of the stomach—the abdominal muscles hard and contracted—no tenderness of the abdomen on pressure—pulse small and feeble—extreme jactitation—great expression of suffering in the countenance. Laudanum was twice given; but the pain continued two hours, and ended in vomiting. He then sunk rapidly, and died at 4 o'clock in the morning.

"*Dissection.*—On opening the abdomen, the stomach was observed to be flaccid and empty, and its contents, which consisted of a large quantity of dark-brown fluid, were effused into the peritoneal cavity, through a ragged opening situated on its anterior surface, and near the œsophageal orifice. The rupture extended from below the lesser arch of the stomach to near its cardiac extremity, and was about four inches in length. The three membranes were not torn equally, the rupture of the peritoneal extending an inch farther than that of the muscular or mucous coat. On the posterior surface of the stomach was a laceration, measuring three inches in length; and there were two or three small ones, from an inch to an inch and a half in length, at its great arch. These lacerations extended only through the peritoneal coat of the stomach, the muscular and mucous tunics remaining perfectly whole. The mucous membrane of the stomach was lined with a great deal of dark-coloured secretion, *beneath which the membrane itself was of a deep red colour throughout,—its texture was softened and partially emphysematous.* The stomach in other respects appeared healthy,—the liver was pale and softened,—the gall-bladder contained a calculus,—the structure of the spleen was unusually soft,—the other viscera were healthy." 450.

Mr. Weekes considers the appearances presented by the inner coat of the stomach as merely indicative of "recent inflammation and *softening* of its texture." But, although *recent* inflammation sometimes produces this softening of texture, *chronic* inflammation much more frequently does the same. We think the history of the case, taken in connexion with the post-mortem phenomena, bears us out in our conclusion, that there was chronic disease of the mucous membrane going on for two years previously to the rupture. The intermissions of vomiting and pain offer no valid objections to this conclusion; for who does not daily see the most unequivocal intermissions of functional disorder, during the progress of fatal organic diseases? These intermissions are not mere exceptions to the general rule. They are positively more common than the reverse—that is, uninterrupted disorder of function where there is disease of structure in any organ. There is hardly a more prolific source of error among young practitioners than this intermission of symptoms. If, for example, a patient who is subject to pain and vomiting after taking food, has intervals of a week or two, without any pain or vomiting, the inexperienced practitioner concludes that there can be no organic disease going on about the pylorus,* he will be very often mistaken. The sensibilities of this organ vary so much at one time from what they are at another, that no implicit reliance can be placed, either on the permanence or intermission of symptoms. In fatal scirrhus of the pylorus, we have frequently observed the sickness and pain cease for whole weeks—but the hardness was still felt on manual examination—the emaciation advanced—and the constipation continued. These and many other examples of the difficulties attendant on diagnosis, should induce the student—yea, and the practitioner, to get into the habit of employing every mean which science and experience have discovered to ascertain the true state of the case, instead of trusting to one or two leading marks, as laid down by systematic writers. These marks are nearly as often false as true.

* "The symptoms, in this case were not such as generally indicate the existence of organic disease; there were considerable intermissions of the symptoms —the patient had enjoyed tolerably good health, and there was no emaciation."— Mr. WEEKES.

When we consider that he was so feeble after the hæmatemesis as to be confined to bed for five weeks, between Christmas and April—that, after that period, "his health had been much impaired," and that the attacks of pain and vomiting had been more frequent than ever, we hardly think Mr. W. is justified in saying these symptoms were not such as indicate organic disease.—REV.

---

### 53. OBSERVATIONS ON THE EFFICACY OF CUPPING GLASSES IN PREVENTING AND ARRESTING THE EFFECTS OF POISONED WOUNDS. By Dr. C. W. PENNOCK.

Dr. Pennock, an American physician, has made an extensive series of experiments in the same line of research with our countryman, Dr. Barry, and has confirmed, in all material points, the facts and opinions of the latter physiologist. His conclusions are as follow.

"*First.* The usual effects of poisoned wounds cannot take place during the absence of the atmospheric pressure procured by the application of cupping-glasses.

"*Second.* Such application does not arrest the deleterious action of the poison by withdrawing it from the exposed surface; on the contrary, the fatal effects are wholly prevented, though not a particle of the substance employed has been abstracted. In proof of this, if a poison in powder, (strychnine or arsenic for instance,) be conveyed by a tube through a narrow wound, in an oblique direction under the integuments, to some distance from the opening by which it is introduced, and there deposited—and under these circumstances the glass be applied over this spot, where the skin is sound and unbroken, the wound being without the bounds of the glass, none of the poisonous substance will be removed, and yet no indication of its action will be presented during the time of the application of the glass.

"*Third* The constitutional symptoms, such as tetanic convulsions, etc. are arrested by the establishment of a vacuum on the poisoned surface; then by removing the poison by an incision through the integuments, the life of the animal is preserved.

"*Fourth.* When the cupping-glass is applied over the opening made in the integuments for the purpose of introducing the tube containing the poison, and this is deposited under the skin beyond the circumference of the glass, none of the effects are manifested during the continuance of the vacuum, but as soon as the cup is removed, the action of the deleterious article commences.

"*Fifth.* If during the application of the cupping-glass, placed as just stated, an incision be made between its edge and the place at which the poison has been lodged, death will ensue as speedily as though the atmospheric pressure had not been removed.

"*Sixth.* If after the application of a glass for a given time, to the sound skin over the spot where the poison has been deposited, the glass be removed, death will then ensue as soon as if no such application had been made.

"This last position is entirely at variance with the observations of Dr. Barry. He expressly says, that "after the glass had been taken off, the animal continued for one or two hours to carry imbedded in his cellular tissue a dose which would infallibly have destroyed him in a few minutes, had the cupping-glasses not been previously applied.

"I have repeatedly observed, however, that if the animal was abandoned to his fate after the glass had been removed, after an application of it for an hour or more, that death took place as soon afterwards as it ordinarily did when no vacuum had been formed. Experiment fifth is in confirmation of this fact."* 13.

The following additional quotation will not be read without interest.

"During the investigations herein detailed, my attention has been forcibly directed to the astonishing effects which are produced by the application of the cupping-glasses in arresting tetanic symptoms:—even where decided opisthotonos has been exhibited, we have witnessed a subsidence of the symptoms, by means of pressure thus applied. I therefore venture to propose it, as one of the means of combating that terrific disease, traumatic tetanus. This variety of tetanus appears to be occasioned by the injury sustained by the sentient extremities of the nerves of the part wounded. It has occurred to me that if these wounded and irritated nervous extremities could be paralyzed, the general spasmodic action, which is produced by the local irritation, would be arrested. *Would not pressure by weights, or ligatures, or the application of exhausted cups over the wound, or in its immediate vicinity, paralyze the sentient extremities of the wounded nerves, and thus suspend, and perhaps remedy, the general spasmodic action?*

"If the constitutional symptoms should be arrested; as a security against their return, *before the cup over the wound* is removed, an incision so extensive as effectually to remove the wounded portion of the nerves ought to be made." 25-

In the course of this investigation, some curious phenomena were observed. Thus, in one instance, having divided the internal jugular vein of a dog, for the purpose of introducing a tube, "jets of blood were observed to issue from the lower portion of the divided vein, at intervals which were synchronous with the expiration from the lungs."

"After this phenomenon had continued for nearly two minutes, the animal experienced great difficulty of respiration, and died very shortly afterwards, manifesting all the symptoms of strangulation. Upon dissection, the right ventricle of the heart was filled with blood, unlike that usually found in that cavity; being red, spumous, and apparently arterialized. The cause of the animal's death was now sufficiently obvious: the atmospheric air had entered the cavity of the heart through the divided vessel.

"Another circumstance connected with the venous circulation, is also worthy of being mentioned. In an unsuccessful attempt to repeat the celebrated experiment of M. MAGENDIE, on venous absorption, one of the platina tubes by which it was intended to form the channel of communication in the femoral vein, was forced so far upwards within it, as to be beyond our reach. Upon searching for the tube, it was found as high as the right auricle of the heart!! This tube was two and one eighth inches in length, and weighed thirty-two grains.

"Assisted by Mr. CHEW, student of medicine, I afterwards introduced into the right femoral vein of a sheep a platina tube, one inch and one fifth in length and weighing nineteen grains. The animal did not subsequently manifest any symptom of pain or uneasiness. He was killed about thirty minutes after the insertion of the tube: upon examination, the tube was not discovered in the entire tract of the iliac vein, nor in the ascending cava, nor even in the right auricle of the heart. It was finally discovered, however, in the larger lobe of the left lung." 26.

In the first experiment, it is evident that the vessel was entirely divided, and, therefore, it can hardly be contended that the platina tube was carried towards the heart merely by the *vis à tergo*. There was something like suction here. The second experiment probably did not comprehend entire section of the vessel, and, therefore, the current of the blood in the vein might not have been completely interrupted.

### 54. CURIOUS CASE OF MONOMANIA IN A MEDICAL GENTLEMAN.

The old doctrine, that mania and mono-

* American Journal of the Medical Sciences, No. 3.

mania are not necessarily connected with any corporeal derangement, begins at last to totter, though still strenuously maintained by many talented modern physicians. As great disorder of function frequently exists without apparent change of structure, these advocates of immaterial insanity argue as did the late Dr. Parry, of Bath—"*de non apparentibus et non existentibus eadem est ratio.*" But this is a very dangerous as well as fallacious rule to go by in pathology. Who has discovered the material change which produces tetanus? Yet who will be so bold as to say that it is a disease independent of material change, though that change is incognizable by the senses?

Dr. Scott, of Cupar, Fife, has lately published a curious case of MONOMANIA, the perusal of which led to the foregoing reflections. The subject was a navy surgeon, (John Anderson, M.D.) who, being placed with many others on half-pay, after the downfall of Napoleon, came to reside in his native town of Cupar. His manners and acquirements caused his society to be courted; but, as he tried several times to settle in practice, and was always disappointed, he became liable to fits of despondency. In 1820, five years after his discharge from the public service, his eccentricities of thought, especially on one particular subject, were remarked by his friends. He had been diving into the mysteries, not to say the absurdities, of animal magnetism, and at last became convinced that he himself was subject to its powerful influence, exerted by some of his best friends. He now became sleepless—or, if he slept, he was the victim of those "terrores magici," conjured up by oppressive and phantastic dreams. Certain individuals could wield at will a malignant influence over him, so as to deprive him of all rest and enjoyment. To escape these *invisibles*, as he termed them, he went to Paris, in the year 1822, but his magnetic enemies soon mingled with the gay crowds on the Boulevards! In the night, these inexorable fiends would press on his breast with the weight of a millstone, disturbing his sleep and locking up his bowels:—At other times, they directed their evil influence to the bladder or rectum, so that he would not have time to undress himself before their contents were discharged. On some occasions, these tormentors would take such unwarrantable liberties with him, that he was forced to roar aloud—and, several times, he applied to the local authorities for protection. On all other subjects, he was perfectly sane —on this point, he was decidedly mad. In process of time, however, he began to evince spmptoms of something more than imaginary hallucination. His memory, in some particular points, failed him. He had the ideas apparently in his mind, but he could not clothe them in words. Having experienced several attacks of pneumonia, he became subject, for several months before he died, to a short dry cough, with severe pain in the back, oppression and tightness across the chest. On the day of his death, he had invited some friends to dine with him; but just as they were sitting down to the repast, their host began to cough up large quantities of blood, and, in a few minutes, he died suffocated.

*Dissection.* There was a large aneurism of the descending thoracic aorta, shewing the usual fibrinous matter, in concentric layers, which had pressed upon the roots of the bronchia, and eroded some of the dorsal vertebræ. This aneurism had burst into the trachea, and thus caused sudden death. In the head, there was found an inflammatory deposit, apparently of long standing, under the arachnoid coat, with thickening of the membrane itself, and adhesion to the parts beneath, about the space of an inch and a half in length, and one in breadth, on each side of the longitudinal sinus, midway between the *crista galli* and the level of the commencement of the lateral sinuses. No other change of structure could be detected.

It would be difficult to determine, in this case, to which of the corporeal lesions the monomania was attributable, though we have little doubt that it was connected with one or both of those above described. We have so often observed the mind to be partially deranged by affections of the heart and great vessels, that we suspect the gradual pressure and destruction of parts around the aneurism to have played an important part in the production of those various morbid feelings by which Dr. Anderson was harrassed, and which were considered to be purely imaginary by his friends. We would not say that the hallucination was the *direct* effect of this disease; but we conceive that this change of structure going on within the chest might very readily disorder the functions of the brain, and disturb the intellect. How far the *locality* of the chronic inflammation in the brain was connected with the monomania or *partial* insanity, we leave for the speculations of the phrenologists. It certainly is very difficult to account for a *partial* disturbance of function in an organ, without supposing a *local* derangement of structure.

---

55. MEMOIRE SUR LA FOLIE DES IVROGNES, OU SUR LE DELIRE TREMBLANT. Par le Docteur LEVEILLÉ.

MEMOIR ON DELIRIUM TREMENS. By Dr. LEVEILLÉ.

[Memoires de l'Academie Royale de Medecine. Tome Premiere.]

Much as has been written on DELIRIUM TREMENS, the disease is every day mistaken, mistreated, and rendered fatal by confounding it with inflammation of the brain or its membranes, to which it certainly bears much resemblance in many particulars. The memoir which we are now to notice, is rather an extended monograph, in the first volume of the Royal Academy's Transactions, and is the production of a man of talent and observation. There are some novel views as well as important facts in this Essay, which are deserving of attention.

1. *Description of the Disease—Action of Alcohol on the Encephalon and Stomach—Cerebral Neuroses, which simulate Delirium Tremens.*

Sometimes cephalalgia, or a tightness and uneasiness at the epigastrium, with vomiting—sometimes a rambling of ideas, or indescribable condition of the intellectual faculties, are the precursory symptoms. But most commonly the delirium bursts forth at once, during or soon after a debauch. It is mild or furious—continued or remittent—and the daily occupations of the individual form the theme of his incoherent and loquacious discourses. The minute symptomatology of this curious disease we shall not detail. The general inquietude, want of sleep, tremor, irritability, quick pulse, white tongue, cool skin, ferretty eyes, incoherent ideas, and perspiration, are the prominent features of the disease.

Of the spontaneous termination of delirium tremens by a return to complete sanity, our author cannot speak. When the disease is simple, and properly treated, it is not mortal, in his practice. If left to itself, it diminishes in intensity, but generally ends in insanity—and this again in fatal apoplexy, presenting, on dissection, a serous effusion in the brain. Opium, in large doses, never failed to cure simple delirium tremens, at whatever period of the disease it was administered. Convalescence was so rapid, that the patients were able, in general, to resume their ordinary avocations in a few days.*

"I have seen (says the author) four patients, in one of whom the disease had lasted three weeks, and was cured in 24 hours, by proper treatment; in the second, the complaint had continued six weeks, and was speedily subdued at last. In another case, where the delirium was of some weeks standing, the patient became calm and rational before he had quite finished an opiate mixture that had been prescribed for him. In all, there could hardly be said to be a state of convalescence—a proof, the author thinks, that the disease was a simple "ENCEPHALOPATHIA NERVOSA," unattended with in-

---

* Delirium tremens has not proved quite so tractable in our experience. Nevertheless it is often cured by opium, if depletion has not been too actively employed. An exquisite case of this disease was lately attended by the writer of this article and Mr. Fincham, of Spring Gardens. The patient (a gentleman) had drunk very hard, principally of brandy, for some months previously, when delirium tremens burst forth, all at once, and in a very unmanageable form. The patient slept not a wink, and it was with difficulty he could be kept from rushing into the streets. He vomited up almost every thing he took, and his pulse was upwards of 120, small and hard. A few doses of calomel and opium brought sleep, allayed the vomiting, and reduced the delirium. We are convinced that one full bleeding would have destroyed this gentleman's life.—REV.

flammation of the brain, its envelopes, or the alimentary canal.

M. Leveillé next enters into a consideration of the question, whether or not the disease may be produced by the absorption of alcoholic molecules, emanating from spirituous liquors, and applied to the mucous surfaces of the respiratory apparatus? Some cases of a doubtful nature are stated, and it is pretty evident that the author does not credit this *modus operandi*. There can be no doubt, indeed, that people may be overcome, and rendered apparently intoxicated by alcoholic vapours inhaled into the lungs; but this is not delirium tremens. The ingurgitation of ardent spirits, however, is another thing. In this case, alcohol is unquestionably introduced into the torrent of the circulation, and applied to the sensorium itself. In the course of fifteen minutes after the injection of some brandy and water into the stomach of a dog, M. Magendie found the blood of the animal exhale a strong odour of the brandy. The muscles, the breath, the sweat, the urine of people who are much addicted to spirituous inebriation, are said to be impregnated with the odour of spirit.

"A cerebral *neurosis* (une nevrose cerebrale) may simulate DELIRIUM TREMENS, and yet be a very different affection, as far, at least, as the *cause* is concerned. I have attended four individuals, who appearantly laboured under DELIRIUM TREMENS, and who were cured by the remedies employed for that disease. On an accurate investigation, I found that none of these patients had had access to intoxicating liquors."

These four cases are succinctly stated; but they are not quite satisfactory—at least the first on the list, who, though reputed a very sober chap, was yet in the habit of drinking *two bottles of wine* daily. It is true the wine was French, on which (as the Irishman says in the play) "it is quite hopeless to get drunk." Yet, we think that two bottles of "VIN ORDINAIRE," are quite sufficient, in certain constitutions, to generate a very strong disposition towards DELIRIUM TREMENS.

2. *Delirium Tremens, complicated with "Determination of Blood," (Coup de Sang) or Arachnitis.*

Are we not (says the author) justified in supposing that inebriation may sometimes cause such a turgescence of the cerebral vessels as may end in arachnitis? If this be admitted, the delirium which succeeds is that dependent on inflammation, and not on that peculiar state of the sensorium, in delirium tremens, which we have supposed to be produced by the circulation of alcoholic molecules in the blood. We have no reason to doubt that the two states may be combined, thus offering a complex disease, presenting opposite indications, and proving extremely difficult to manage. M. Leveillé relates two cases shewing a continuance of delirium after the removal of all signs of cerebral turgescence by depletion. We shall just glance at these.

*Case* 1. A man, aged 55 years, gave himself up to intoxication, in consequence of some domestic troubles. After a bacchanalian orgie, he had an epileptiform attack. Next day (28th May) a mustard pediluvium was ordered, and a dozen of leeches to the sides of the neck. The leech-bites bled profusely—all symptoms of cerebral compression vanished—but delirium came on. 30th. Severe cephalalgia—face red—conjunctivæ injected—eye-lids moist and swelled—violent irritability—refusal of drink—strong pulsation in the carotids. A deep seton having been introduced in the nucha, there was a profuse hæmorrhage from the part. Ice was applied to the head. In the evening the patient was calm and pale. The strait-waistcoat was removed; but the patient soon attempted to escape, and was secured. 31st. There was delirium when fatigued by questions. Two tepid baths. In the evening he was calm; but in the night he was furious. 1st of June, took two grains of tartar emetic, which vomited him. He was alternately calm and delirious. On the 2d of June, the delirium entirely left him, and he rapidly recovered.

*Case* 2. A servant, aged 33 years, strong and athletic, got drunk on the evening of the 28th May, and went to bed in that state. Next morning he was found apoplectic, and lying on the floor of his room. He was bled from the foot. On recovering from the apoplectic seizure he became delirious, and even furious. This state continued till the 21st of June, when he was received in the Maison de Santé. An emetico-cathartic mixture was exhibited next morning, and operated well. In the night he was more than usually unruly. 23d. Eye fixed and prominent—face flushed—eye-lids swelled—conjunctivæ injected—tongue red, and moist—pulse quick, full, and hard—abdomen contracted—constipation—urine copious—constant delirium and agitation. A deep seton was inserted in the nucha, and bled most plentifully. 24th. Less delirium—the pulse reduced. Ten leeches to each side of the neck—two warm baths. He had some sleep in the course of the day, and the bowels were opened. 25th. Tongue red at the sides and coated in the middle—pulse small and feeble—tetanic rigidity of the flexor muscles of the wrist—great agitation at night. 26th. Escaped from his bed, but was secured. In the evening he was rational. 27th. The delirium has returned. In the course of two or three days more the mind became tranquil, though there was an astonishing emaciation of the body. He was discharged cured on the 7th July.

It is remarked by the author that, in both these cases, the delirium was rather increased than relieved by the sanguineous depletion which must have taken off the turgescence of the vessels of the brain—and hence he naturally concludes that the delirium in this disease is not the delirium of meningitis. Nevertheless the two states may co-exist, and form, in fact, a very troublesome complication.

3. *Delirium Tremens complicated with Gastric or Enteric Irritation or Inflammation.*

The school of Broussais on the Continent attribute the affection of the sensorium, in this disease, to sympathy with the stomach or bowels. The disciples of Abernethy would say the same in this country. Nevertheless, our author can only see a coincidence, and not a cause in the gastro-enteric irritation. There can be no doubt indeed that the stomachs of drunkards are in a bad condition; but if delirium tremens depended on derangement of the digestive organs solely, we should have the disease much more frequently in practice than we now have. A great number of cases are related by M. Leveillé, to shew that the cerebral and abdominal disorders are independent of each other; but these we need not notice in this place. The following are the conclusions to which our author has come.

"The mania of drunkards (*mania a potu*) is no other than delirium tremens"—excess of inebriating potations is the efficient cause.

"This delirium consists in exaltation of the vital powers of the brain, excited by alcoholic molecules absorbed from the surface of the stomach and bowels, and carried into the torrent of the circulation. It is very doubtful whether or not these molecules, applied to the air-passages, are capable of producing such an effect. It is also very doubtful whether the disease has ever taken place without the application of ardent spirits, or other intoxicating drinks.

"There are some cases on record, and some of the first cases treated by myself, where there seemed reason to believe that the delirium resulted from determination of blood to the head—from acute, or from chronic meningitis; but as the general cause was applied, in these cases, I am persuaded that the delirium was independent of the congestion or inflammation about the brain.

"The disease assumes a chronic form in some elderly people, who generally die apoplectic and maniacal at the same time; there are found, on dissection, serous effusion, thickening of membranes, and other appearances usually remarked in the brains of the insane.

"The complications of this disease with gastritis and entero-gastritis are very frequent. The mental hallucination is the first symptom which is perceived:—it gives way to opium; but the gastro-enteric affection pursues its course. They are independent complaints."

---

56. Case of Catalepsy, combined with Mania. By Dr. Burrows.

We remember hearing a remarkable and highly interesting case of catalepsy combined with mania, read at the Medico-Chirurgical Society, about three years ago, and wonder much that it was not published, seeing that many cases of far inferior interest have seen the light through that channel. On looking over Dr. Burrows's late work on insanity, we recognized the case, and find that the patient had been in Dr. B.'s asylum while afflicted with her distressing malady. As it involves some important etiological and pathological considerations, we shall condense its most important features in this article.

*Case.* A young female, of education above her situation in life, (which was that of a house-keeper,) after being tempted to live in concubinage, had the proffer of marriage, provided the ceremony took place the very next day after the proposal. The agitation of mind, on this occasion, brought on a premature eruption of the catamenia, and in this state the marriage was solemnized, though with great reluctance.

"The new-married couple set off in the evening, to travel in a stage-coach to the place where they were to sleep. During the journey her passions were highly excited, and subsequent intercourse was attended with much pain. After having slept about an hour, she suddenly awoke in a violent alarm, saying, she had had a frightful dream, and then complained of a dreadful pain in her head. Presently she jumped out of bed and flew to the window, which her husband fortunately prevented her from opening; she then for a short time was unconscious of all around her, and fainted. On recovery, she became delirious and furious. The catamenia ceased from this time."

The medical practitioner who was called in, bled, purged, leeched, blistered, bathed, and starved the patient—and, in about three weeks, the symptoms gradually abated. A visit from her husband, at this juncture, together with indiscriminate communication with friends, produced a relapse, and mania, though in a mild form supervened. This subsequently changed to melancholia. In a fortnight after this she was removed to Dr. B.'s establishment. Her countenance was sullen and pallid; the eyes heavy, turgid, and cast downwards; the tongue foul; bowels inert; the pulse rather full and slow; the surface of the skin, and especially the extremities, below natural heat. She answered few questions, and those only in monosyllables; and she was very averse from moving. The patient was observed to make frequent pressure on her head, and the carotid arteries beat stronger than any others. The sinciput was hotter than natural, and the extremities were cold. The head was shaved—the occiput cupped—and cold lotions applied to the vertex. The bowels and stomach were cleared by purgatives and emetics. This was on the 10th November. On the 24th, some ptyalism came on from calomel that had been taken; and all the symptoms were greatly ameliorated. On the 8th December the ptyalism ceased, and all the bad symptoms speedily returned.* On the 18th December we find her assuming the cataleptic character.

"She preserves the exact posture, whether lying, sitting, or standing, in which she is placed; eats mechanically whatever is put into her mouth; if spoken to sharply, the only notice is a sardonic grin. The skin resembles white wax or marble, and is again colder than natural; feet very cold; pulse feeble; respiration undisturbed and scarcely perceptible; eyes fixed and turned upwards; alvine dejections natural; sleeps well, and when taken up in the morning, is dressed like a helpless infant."

There was little change during the next fortnight. During the cataleptic paroxysms, the carotids were observed to beat with great quickness and strength, while the pulse at the wrist was feeble and slow. This circumstance is utterly unaccountable, and nothing but the assertion of Dr. B. would induce us to believe it. If we saw the fact ourselves we would distrust the evidence of our own senses.

On the 1st January all the symptoms were suddenly aggravated. She became a perfect statue; sensation and volition were quite suspended; the evacuations were involuntary; there was a constant sardonic expression in her features; mouth open, and a large quantity of saliva flowed unrestrained; the eyes were immovable, and embedded in the upper eyelids; every limb retained the position in which it was placed; even the most

* We think Dr. B. might have taken a useful hint from the above accidental occurrence. We should have been inclined to keep the patient under the influence of mercury for some weeks.—*Rev.*

painful was endured without any apparent suffering, and that for a space impossible to be preserved by any one in health. She resisted every attempt to rouse her by moderate pinching and pricking. These paroxysms, varying in intensity, lasted through the day. She now exercised but one voluntary animal function—deglutition. Various remedies, including local depletion from the head and spine, were employed, but with little effect. On the 12th February, "she arose in the morning in possession of every faculty, both corporeal and mental." She voluntarily assisted in domestic affairs, and talked rationally. She had a perfect recollection of all that passed prior to the attack of catalepsy—all since was a blank. It was mortifying to find her torpid and mute the next day. She was cupped and vomited—and a seton was inserted in the nape of the neck. At this period she removed from Dr. B.'s asylum, but he learnt that she derived very great benefit from the seton—that the menses re-appeared—that she perfectly recovered—and has since borne several children.

"Many circumstances in this case indicate determination of blood to the brain: the interruption of the menstrual flux, the discordance between the force of the carotid and radial arteries, and the temporary relief always produced by abstracting blood by cupping from the head during the existence of the cataleptic symptoms, support this inference." 191.

We believe there is not a case on record which more distinctly shews the operation of moral causes in deranging the corporeal, and through them the intellectual functions. The case is altogether very remarkable and instructive.

---

### 57. HYDROTHORAX.

No disease is more common in the mouths of medical practitioners than HYDROTHORAX—none is more rarely to be found, as a primary or original disease. But this is not the worst part of the business. In three-fourths of those cases denominated hydrothorax there is no water in the chest at all, but merely an inflammatory engorgement of the lungs, to be cured by the lancet rather than by squills and digitalis. In confirmation of these remarks, we shall here introduce an extract from a lecture lately delivered by Dr. Stokes, to the students at the Meath Hospital, Dublin.

"Before I speak of the latent affections of the lung, I shall allude to an application of the Stethoscope, as important, perhaps, as any of those I have described. Every one has heard of *hydrothorax,* or *water on the chest.* The disease and its symptoms have been described in the different works on the Practice of Medicine; nay, further, books by grave and learned men have been written on this disease; cases have been detailed, and dogmata inculcated. Corvisart was the first who shewed that idiopathic hydrothorax was a disease of extreme rarity, and that when serum was found in quantity in the cavities of the pleura, we should not consider its accumulation as a disease, but rather as the consequence of some antecedent affection of the lungs or heart. The immortal Laennec has further established this proposition, and has proved to demonstration, that hydrothorax can scarcely ever be considered as a primary disease.

"It is as amusing as it is extraordinary, to find, that even now, when so many years have elapsed since the publication of the work of Corvisart, many medical men are to be found who would consider this statement as absurd, who still continue to be guided, or rather misguided, by the writings of Cullen and Maclean, who consider every case of cough, difficulty of breathing, lividity of the face, orthopnœa, scanty urine, and swollen extremities, as hydrothorax, when, in fact, a moment's application of the Stethoscope would show that they were mistaken.

"Many of these cases, as has been stated in the Report of the Hospital by Dr. Graves and myself, have been sent into us as cases of hydrothorax, but in no *one* instance have we found this statement correct. Many of them we might, indeed, state, had serous effusion in every part of their body, save and except in the cavities of the pleuræ. Some of these cases were instances of chronic bronchitis, terminating in that dilated state of the pulmonary cells, called emphysema by Laennec; others were cases of this affection, complicated with disease of the heart; but the great majority were cases of chronic pneumonia, mistaken and maltreated from the very commencement; mistaken in consequence of men adhering to the definitions of the older writers, and maltreated by their trusting to the diuretic plan, and neglecting to overcome the disease in the first instance. By the Stethoscope such cases can at once be detected, and distinguished from hydrothorax. As I stated in the former part of this lecture, whenever effusion takes place into the cavity of the pleura, the sound of respiration becomes diminished, and ultimately ceases in the affected part.

"We have had many patients in whom after death, a large quantity of fluid was found in the cavities of the pleura; yet we know by the Stethoscope that this effusion did not take place until within a few hours of the fatal event. The patients died with hydrothorax, but not of it. This important fact, discovered by means of the Stethoscope, has thrown a new light on such cases, and has enabled the physician to save life, where, without this knowledge, it would be inevitably lost.

"Observe now what an important error you may fall into by trusting to the descriptions given by the older writers. You get a patient with dyspnœa and orthopnœa, swelled legs, scanty urine, and every other symptom of hydrothorax, according to Maclean, Cullen, and Good, and you commence giving him squills, nitrous ether, and digitalis, and your patient gets worse and worse, till at length you apply the Stethosope, and find that there is no hydrothorax at all, the disease being, in fact, pneumonia, and calling for the employment of the lancet and other antiphlogistic means. These, Gentlemen, are undeniable truths, which many of you must have seen exemplified again and again in our wards. Many of the cases recorded by Maclean are nothing but chronic pneumonia, proved, in fact, by his own dissections."

We recommend the foregoing observations to the attentive consideration of those LIGHTS OF THE PROFESSION, who *are*, or rather *have been*, perpetually sneering at the stethoscope, as a bauble —an "inutile lignum"—a piece of quackery, &c.

---

58. SERIES OF CASES EXEMPLIFYING THE INFLUENCE OF ERGOT OF RYE ON THE HUMAN UTERUS. By W. MICHELL, M.R.C.S.*

Mr. Michell appears to have given the ergot of Rye a far more extensive trial than any English Practitioner, and as the results of his experience are detailed in a very unostentatious form, and bear intrinsic marks of authenticity, we shall, in this paper, offer our readers a condensed view of the facts contained in the 10th chapter of the work—a chapter dedicated to the narration of 31 cases.

*Case* 1. Mr. M. was called to a labour at 2 A. M. and found the os uteri the size of a shilling—the pains had been quick and grinding, but now easy. He waited ten hours, and then infused half a drachm of ergot of rye in four ounces of boiling water. This was taken at ten minutes past noon. In 14 minutes she had a pain, and exclaimed—"clap a hand, or the child will fall to the ground." The head was found on the perineum, and the child was born before the pain was over. This was the woman's 12th child. The placenta was expelled before Mr. M. could tie the umbilical cord. The child was living.

*Case* 2. To this labour he was called at 1 A. M. It was her sixth child. The pains had nearly left her—the os uteri was not dilated—and the labour had apparently gone off. Mr. M. waited seven hours, and then gave the ergot. In fifteen minutes a pain came on—in 34 minutes from the exhibition of the medicine, the child was born. The dose was half a drachm, as before, in infusion.

*Case* 3. This was a lingering labour, and Mr. M. gave the ergot, as above, but it failed; and in 20 minutes the dose was repeated. In a quarter of an hour after this, she had sharp pains. In half an hour, they were very strong—the os uteri was the size of a crown-piece. In two hours delivery was effected. The child had been evidently dead for some time.

* On Difficult Parturition.

*Case* 4. This was also a lingering labour. After repeated visits Mr. M. found her with pains, but the os uteri was not larger than a sixpence. Half a drachm of the ergot was infused, and given with a third part of milk. In 13 minutes the pains were altered in character. In half an hour, the os uteri was dilated the size of a wine-glass, and the pains were expulsive, and continued till the child was born.

*Case* 5. Mr. M. was called at 5 in the morning—pains severe—no dilatation of the os uteri. These pains, apparently expulsive, continued all the day, and opium was twice given. At 12 o'clock the os uteri was still undilated—the same at 8 in the evening. It was her first child. The ergot was sent for, and administered at midnight, in the ʒss. dose. In 18 minutes the woman put on the Hippocratic face, and exclaimed that "her bowels were cutting in twain." At half past 12, the os uteri was found a little dilated—at 1 o'clock, it was the size of a wine-glass—at half-past 1 the head bore firmly on the perineum—and delivery was effected in two hours and eighteen minutes after taking the ergot. No flooding or any disagreeable symptom followed. One ounce of brandy is always given by Mr. M. immediately after delivery.

*Case* 6. Mr. M. examined a female at 4 o'clock, p. m. and found the os uteri undilated—the waters broken some hours previously—the vertex presenting—pains slight. Foreseeing that the case would be lingering, he gave her ʒss. of the ergot in infusion. This was at 5 o'clock. In 14 minutes a smart pain came on, and was succeeded by others without intermission. In 40 minutes, the os uteri was the size of a shilling, but firm. In 90 minutes, it was the size of half-a-crown—in 2 hours and a half it was the size of a tea-cup—and in 40 minutes more, the delivery was effected.

*Case* 7. Mr. M. was called to a case where the face presented, the pelvis being capacious. By way of experiment, the ergot was administered. The medicine quickened the pain in a quarter of an hour. In an hour, the pains were excessive—the os uteri fully dilated—but the uterus was unable to propel its contents. In three hours, a second dose was administered. This again roused the uterine action, which continued for an hour, but without effect. The child was then turned and delivered. The child was dead.

"Here an objection may be taken to turning, after Ergot has been administered, but I found it not at all more difficult than usual. Again, it may be surmised by those who are adverse to the use of Ergot, that this child was killed by the *poisonous effects* of the Ergot, and as the whole case affords the strongest possible refutation of such a supposition, it was fortunate that I was induced to make the experiment which I have just related. On laying my hand on the lower part of the Abdomen, every part of it appeared as full and tense as before the delivery, and on passing up the finger I found on examination another Child in utero. As the pains did not come on, I hesitated whether to have recourse to turning or to give another dose of Ergot, but soon decided in favour of the Spurred Rye, of which I administered ℈ii. In five minutes it had its usual effect, and in nine minutes and a half a fine healthy child was pushed into the world. Now this was five hours and fifty minutes from the first administration of the Ergot, and one hour and forty minutes from the second, and nine minutes and a half from the last dose; in all, this patient took two drachms, wanting one scruple. Now the death of the first child would certainly have been attributed to its being ergotised, and thus poisoned. But I consider it a very fortunate answer to those who suppose still-born children to have been injured by the Ergot. Such a notion has arisen merely from the circumstance that Ergot is seldom given except in cases in which the children are frequently dead born, either from laborious accouchement, or from the pressure on the child's head. It may be objected, and with great propriety, that I ought not to have given the Rye at all in this case, but I was desirous of observing its effects, if allowed to exhaust itself, and whether any injury would result from it in wrong presentations, and from this case we may observe, that it exhausts itself in about three or four hours." 91.

*Case* 8. Called at 7 o'clock, A. M.—

grinding pains—but no progress. These pains continued more or less, from the 12th of February till the 7th of March. At 8, A. M. the pains were severe. On examination, the os uteri was found to be long and soft, not open enough to admit the finger—the water dribbling away. Mr. M. remained all night. At 3, P. M. next day, the os uteri was still undilated. Opiates had been several times given, without any good effect. At half-past 3 gave the ergot. At 4, the pains were very severe and cutting. At 20 minutes past 5 the os uteri was dilating—at 6 the head was forced on the perineum—at a quarter-past 7 delivery took place. The child was dead.

"The head was elongated eight inches and a quarter in length, and three inches in circumference. Had not the child died I do not think the delivery would have been effected without the aid of instruments, as the head, had it been living, would not have been moulded into its peculiar shape. This woman was forty-five years of age, had been married twelve years, but never pregnant until this occasion. In this instance the Ergot was given after waiting many hours without the least hope of a conclusion in less than forty-eight hours; the circumstances were certainly very unpromising, for its having a good effect, but here its influence on the Uterus was greater than I expected, the delivery being effected in three hours and three quarters, and without doubt greatly accelerated by the Ergot." 94.

*Case* 9. Called to a woman at 9 o'clock in the morning. Throes strong, and, to appearance, expulsive—os uteri undilated. These pains continued till 4, P. M. when the os uteri was still close—the cervix uteri not obliterated. Half a drachm of the ergot was given. In 13 minutes, the throes were more expulsive. At 5, the pains were constant—at half-past 6, delivery. This was a first child.

*Case* 10. Sent for at 1 o'clock, 5th May, 1818—pains smart, but with intervals of an hour. At half-past 2 gave the ergot. In eight minutes there was a pain —another in twelve, which continued five minutes, "when the child was expelled to the astonishment of all present."

"This case I marked as particular, as the Os Uteri was dilated only about the size of a shilling when the Ergot was given, and the delivery was effected before any thing could be procured to tie the cord." 96.

*Case* 11. Mr. M. was called on the 26th of June, 1827, to a woman in labour of her eleventh child, at half-past 8 o'clock. Waited till 10, the pains being very slight, during which the waters were discharged. At 20 minutes past 10 the ergot was given. At 40 minutes past 10 the throes were severe. On examination, it was found to be a breech-presentation. In 47 minutes the child was born alive and well.

"Had this child been born dead, as is frequently the case in unnatural presentations, the Ergot would have been again condemned. I have given this case, although it contains nothing particular beyond scores I could record, except that it was a breech presentation, and that all her other labours had been lingering. As I had not attended this patient before, I was not aware of this circumstance, or I would not have waited two hours. These cases I consider quite sufficient to explain the modus operandi, and to establish the utility of Ergot." 104.

So think we. Our obstetric brethren will not, we think, be slow to have recourse to this valuable accelerator of parturition. If it maintain the character which Mr. Michell has given it, the practice of midwifery will lose more than half its drudgery, and medical men will save an immensity of time into the bargain!

---

### 59. Yellow Fever at Plymouth.

Thirty years ago the above heading to an article would have startled John Bull from Dover to the Orkneys, and put Plymouth in quarantine. But the dread of contagion is now much subsided, and the "Bulam fever, the yellow fever, the "NOVA PESTIS" no longer excites terror, even in the non-professional mind. It is not often, however, that a fever answering so completely to the dreadful transatlantic scourge, as the following, is seen on our gloomy shores. It is recorded by Mr. Niell, surgeon of His Majesty's ship Britannia, a gentleman well acquainted with West India diseases.

The subject was messman to the officers of the ship—of sanguine temperament—addicted to eating and drinking freely—and exposed to the vicissitudes of the seasons. The summer of 1826, was almost tropical in this country, and, on the 8th of July, the patient was seized with rigors, succeeded next day by violent pain in his back, head-ache, and great prostration of strength. He did not apply for assistance till the morning of the 11th, when there was high fever, and 28 ounces of blood were abstracted and produced syncope. There was no inflammation evinced by the blood. 12th. Much nausea and vomiting—*pain in the right hypochondrium*. To take calomel every four hours. 13th. Nausea—skin of face and neck turning yellow—burning sensation at the pit of the stomach, and in the direction of the œsophagus. 14th. Nausea distressing—yellow tinge of the skin deeper—singultus—slight coma. Calomel every two hours. 15th. Black vomit resembling coffee grounds—skin of a deep yellow—pulse 120—singultus—bowels purged. Took a bottle of sherry and half a pint of brandy, with ether, during the last 24 hours. [What will Broussais and his disciples say to this treatment of intense Gastro-Enterite? It will bring forth volumes of criticisms in France!] Ten grains of calomel and four grains of carbonate of ammonia every four hours. 16th. Had some sleep this morning—singultus and sickness not quite so harrassing—yellow colour of skin very deep—face much flushed—bowels relaxed, the motions being black—tongue and mouth covered with black sordes. "*Wine, brandy, ammonia, calomel, and æther continued.*" 17th. "Burning sensation in the stomach and œsophagus has disappeared." Pulse 110—singultus still troublesome—alvine discharges of a lighter colour, containing "floating black flaky substances." Same medicines continued. 18th. Mouth sore—gums swelled—pulse 100, regular and full—bowels open—evacuations of a dirty brown colour. Same medicines. 19th. Pulse 96, full and rather hard—skin soft—yellow suffusion still deep—some good fæcal matters in the stools—singultus less—coma increased—takes soup and sago in pretty large quantities. Brandy and part of the wine discontinued—calomel, ammonia, and ether continued. 20th. Same as yesterday. A bottle of wine in 36 hours—medicines continued. 21st. Yellow colour less intense—gums, cheeks, and tongue ulcerated—bowels open—stools of a dark green colour. Same treatment. Ptyalism did not begin to flow till the 27th—when we find the skin cool and soft—the evacuations of a dark bilious hue—mercurial odour on the breath strong. By the 31st. the tonic treatment was commenced, and the patient slowly recovered.*

*Remarks.* The above case is a very interesting one. It was decidedly an example of "yellow fever"—at least it had every attribute of that terrible malady—and so far it is capable of exciting some important pathological considerations. In a therapeutical point of view, it will probably occasion some difference of opinion. In politics, a measure is treasonable or loyal, just as it happens to be successful or the reverse. So, in medicine, we suppose we must consider that mode of treatment the best, which ends in recovery. Knowing the talents and experience of Mr. Niell and Dr. Dickson, who had the charge of the case, we should be extremely loth to differ from them in therapeutics; but, judging from the record before us, we shall not offend by saying that we should have pushed local depletion farther, and been less liberal of mercury and wine. That the gastro-hepatic system bore the great onus of disease, cannot be doubted, and that all the aggravated symptoms were produced by the remedies employed, will be confidently averred by our Continental brethren. In this sentiment we do not join; but still we think that the majority of practitioners will agree with us in the opinion which we have broached, as to the non-necessity for such powerful stimulants and mercurials as were employed—and as to the propriety of applying numerous leeches to the region of the liver and stomach. It is much more easy, however, to criticise a case when it has terminated, than to treat it at the bed-side; and therefore we are perfectly ready to grant that the talented practitioners in attendance (both of whom we are proud to call our friends) acted in all respects with wisdom and skill, according to the symptoms that presented themselves from day to day.

* Med. and Phys. Journal, Aug. 1828

### 60. HORÆ LACONICÆ.

### No 3.

### DYSMÆNORRHŒA.

The sooner that the laws of *vitality*, in physiology, are abolished, and the laws of *mechanics* or *physics* established in their stead, the better for THERAPEUTICS! Dr. MACINTOSH has lent a powerful hand to this good work, and deprived the College of Physicians of one more of their *monopolized* articles — DYSMÆNORRHŒA. This is no longer to be considered within the domain of physic—but of surgery. There will be no more EMMENAGOGUES prescribed. The BOUGIE is to supersede aloes and myrrh! "It *always* occurred to me," says Dr. M. "that there was some *mechanical* cause which produced dysmænorrhœa." It is refreshing to see a man of genius *come* to an investigation with a hearty *predetermination*. These are the men who are sure to overcome difficulties —and as there is a whole tribe of troublesome diseases characterised by *dys*, or difficulty, it is most fortunate that Dr. M. has undertaken the management of one of the most intractable of them all. We see stricture of the urethra produce dysuria—and a bougie cure the DYS:—Why, then, should not stricture of the os uteri produce dysmænorrhœa; and why not cure it by the bougie? The idea is so natural, the conclusion so obvious, that it is wonderful it should not have been entertained long ago. Yet so it was, that even Dr. Macintosh did not hit on the precise mechanical cause till the year 1823, when a gentleman presented him "an uterus *without an orifice*." A new light now beamed on this dark subject. If the want of an orifice prevented the menses from flowing at all, why should not a small orifice produce difficult menstruation? "Mechanical dilatation appeared to be the remedy;" but Dr. M. hesitated to propose it to modest women. At length, a young woman came from the country, who, up to the age of 16 years, had been healthy; but from that till the age of 22, fell off in health. She had never menstruated, but had pains and sense of weight in the loins monthly. On examination, no orificium uteri could be felt. A very small silver probe was attempted to be introduced, but without success. The sharp triangular extremity of the same instrument was next made to perforate into the cavity of the uterus, and the button point afterwards passed up to the fundus. A good deal of irritation succeeded, and the operation could not be repeated till the eighth day, from which time it was persevered with till a male bougie, No. 6, was got in. This was followed by much local and constitutional derangement, and, in two days after this tumult in the system, the patient menstruated.

This was a case of complete *occlusion*, and how far it bears on the subject of dysmænorrhœa, we leave to our obstetrical brethren to determine. Dr. M. informs us that he has employed the bougie in six or seven cases, not detailed, with uniform success—and we are bound to believe him. But we are not bound to adopt either his principles or his practice. We firmly believe that the *former* are as erroneous, as the *latter* would be dangerous, if generally pursued. Let those who have seen most of female diseases reflect on the numerous moral and physical causes which disturb menstruation, and render it either painful or null—let them, then, ask themselves what proportion of dysmænorrhœas are attributable to mechanical stricture of the os uteri—and what to other causes. Probably there may be as many as there are cases of dyspnœa, dependent on stricture of the rima glottidis.

### SUCCESSFUL EXCISION OF THE CERVIX AND OS UTERI. By Dr. WEATHERILL, of Liverpool.

Of all the cases which LACON has ever read, that of Dr. W.'s is the most remarkable—for ignorance of language, confusion of thought, and defiance of all credibility. "*Ex ore tuo te judico.*" "A female, aged 37, applied to the DOCTOR, (one of Dr. Harrison's class, we presume,) for the cure of leucorrhœa, excoriation, and "symptoms of disease about her loins and *lower abdomen*," attended with "feelings of *bearing down* about the *upper and inner part of the thighs*." The discharge was exceedingly troublesome

—" being sometimes watery and *excoriated*."* On examination, the Doctor found a world of mischief—enlarged and indurated os uteri—evidence of excavations—ulcerations—jagged and irregular edges, &c. &c. On the 12th of May last, this unfortunate patient was placed on a table, and secured as for lithotomy. The steps of the operation shall be given in the Doctor's own words—and we venture to assert that such a description is without a parallel in the annals of surgery.

"Two dilators, made with short handles, and blades set at right-angles, about six inches in length, with edges and ends rounded and quite blunt. These were introduced, and held by assistants, to obtain lateral dilatation of the vagina. With a tenaculum, strong and of large dimensions, I transfixed the cervix and made extension. The uterus, however, was not to be drawn down so easily, and every attempt to do so sufficiently to proceed with the knife, *in situm*,† entirely failed, and caused extreme agony to the patient; and it was not until I had fixed the tenaculum through that part of the vagina which attaches itself round the cervix, and laid hold of the body of the uterus, using at the same time forcible extension, that I was enabled to see the seat of disease. The os uteri, internally, was nearly destroyed by ulceration; the cervix contained several small ulcerated openings, as if eaten by worms, with inflamed margins, and the body of the uterus I believed to be more or less affected. Guided by these appearances, I hesitated not to extend my incisions beyond the grasp of the tenaculum, which of course brought me into the cavity of the abdomen. Before the operation was more than half completed the hold of the tenaculum gave way, and the intestines protruded; which, added to the shrieks of the patient, rendered my situation rather perplexing. I made several fruitless attempts to secure and draw down the uterus again with the tenaculum, and was compelled, after some delay, this instrument being of no other use than merely to keep the uterus steady, to finish the operation in the vagina. After the vagina was partially filled by a plug of linen, the woman was put to bed; and, although she had not lost more than ℥viij. of blood during the operation, she appeared much exhausted. I ordered a table spoonful of port wine, to be given frequently, and 20 or 30 drops of laudanum, if in great pain. I think it needless to enumerate her situation and treatment from day to day; it is sufficient to say, that she recovered without the intervention of one bad symptom, and took no other medicine than a few doses of a common cathartic mixture."

We are inclined to be charitable on this occasion, and to believe, either that the Doctor has hoaxed the Editor, or that the whole was a dream—an "unsubstantial pageant"—very well calculated to make the "vulgar stare." If there exists such a personage as Dr. Weatherill, we are quite confident that he *would have hesitated* (though he says he did *not* hesitate) to "extend his incisions beyond the grasp of the tenaculum," so as to lay open the cavity of the abdomen, and let the intestines come into open day! How he contrived to finish the operation he gives us no information—probably because he awoke from his dream in the midst of the chaotic imagery already delineated! After such a painful recital, it is gratifying to find that the patient has—"ventured across the Atlantic," and pitched her tent—

"Where ANDES, giant of the Western Star,
With meteor standard to the winds unfurl'd
Looks from his throne of clouds o'er half the world."

We will venture to assert that, of all the *wonderful* things which Mr. Giant Andes must have seen, during his long spectatorship, this case of Dr. Weatherill's is the most wonderful. We question whether even Brother Jonathan will believe it.

### Dr. Blundell's Extirpation of the Uterus.

A still more formidable operation than

* It was reserved for the nineteenth century to see medical journals conducted by men who had no knowledge of *practical* medicine! Will it be believed, in future ages, that a *physician* could write, or an editor (in the simplicity of ignorance) print, such palpable absurdity as an "*excoriated* discharge"—a fluid with its skin off!!

† "In *situm*"! Where is Lawyer Keene, who corrects the bad English and worse Latin.

the above, and, we dare to say, an operation more cautiously and scientifically performed, remains to be put on record.

The patient was a married woman, aged 50 years, and had borne children. She had laboured for some time, under offensive and extremely profuse sanguineous discharges from the womb which, on examination, was found to be in a state of ulcerated carcinoma. The organ was moveable—the size of a goose's egg—its mouth broad, open, and of cartilaginous hardness—with an ulcer on the surface of the diseased mass, the size of a shilling. There did not appear to be any visceral disease, and, although the woman was greatly debilitated, from the loss of blood and other effects of this terrible disease, she was deemed a subject for an operation which may fairly be deemed the *most formidable* to which the human frame has submitted, or modern surgery dared to achieve. The steps of the operation are minutely described by Dr. Blundell; but they cannot—they ought not to be abbreviated.

"The bowels having been cleared, and the patient being resolved to submit to the operation, on the 19th of February, 1828, I determined to remove the diseased parts without further delay. For this purpose, having placed the woman in the obstetric position usual in this country (on the left side I mean), close upon the edge of the bed, with the loins posteriorly, the shoulders advanced, the knees and bosom mutually approximated, and the abdomen directed a little downwards towards the bed, I began the operation.

"*First Stage of the Operation.*—I commenced by passing the index and second finger of the left hand to the line of union between the indurated and healthy portions of the vagina; the finger being converted into a cutting instrument (varying with the exigencies of the operation), by means of a moveable knife, which requires a word or two of description. The blade of this knife, not unlike that of a dissecting scalpel, was mounted upon a long slender shank, which, including its large handle, was about eleven inches in length; and with this stem the blade was united, so that its flat, or plane, formed with the stem an angle of 15 or 20 degrees. The first and second fingers of the left hand then being in the back of the vagina, contiguous to the diseased mass (as before observed), by taking the stem-knife in my right hand, I could at pleasure lay the flat of the blade upon the front of these fingers, and urge the point of the instrument a little beyond the tip. The apex of the fore-finger being in this manner converted into a cutting point, by little and little I gradually worked my way through the back of the vagina, toward the front of the rectum, so as to enter the recto-vaginal portion of the peritoneal cavity, frequently withdrawing the stem-scalpel, so as to place the point within the tip of the finger, and then making examination with great nicety, in order to ascertain whether the vagina was completely perforated, minute care being necessary in this part of the operation to avoid wounding the front of the intestine.

*Second Stage of the Operation.*—A small aperture having been formed in this manner, in the back of the vagina, through this opening the first joint of the fore-finger was passed, so as to enlarge it a little by dilatation and slight laceration (safer than incision). This done, and a cutting edge being communicated to the finger, by placing the plane of the blade in such a manner that its incisory edge lay slightly advanced beyond the side of the finger now lying in the aperture, after drawing the point of the instrument within the tip of the finger, which operated as a guard, I proceeded to make an incision through the vagina transversely, that is, in a direction from hip to hip: for this purpose carrying the finger with its cutting edge, from the opening in the vagina already made, to the root of the broad ligament on the left side, so as to make one large aperture. I then took a second stem-scalpel, formed on the same model as the preceding, with this difference, that the incisory edge lay on the other side of the blade; and laying this instrument on the fore-finger as before—in such a manner, however, that the cutting edge lay forth on the other side of the finger (to the right of the pelvis, I mean), I carried the finger thus armed from the middle of the vagina, where the former incision commenced, to the root of the broad ligament on the right side; so that, at the end of this, which was the second step of the operation, the diseased and healthy portions of the vagina behind became completely detached from each other, by a transverse incision, which stretched across the vagina, between the roots of the broad ligaments immediately below the diseased parts. At this time the intestines could be felt hanging about the tips of the fingers; but the blade of the scalpel lying on the finger, in which it was as it were imbedded, the risk of a wound, whether by point or edge, was completely prevented.

"*Third Stage of the Operation.*—The back of the vagina, then, having been divided in this manner, I urged the whole of the left hand, not of large size, into the vaginal cavity—and the more easily because the woman had borne children; afterwards passing the first and second fingers through the transverse opening along the back of the uterus—this viscus lying, as usual, near the brim of the pelvis, with its mouth backward, its fundus forward, and a little elevated just above the symphysis pubis. This manœuvre premised, under full protection of these fingers, now lying between the womb and the intestine, taking a double hook, mounted on a stem eleven inches long, I passed it into the abdominal cavity, through the transverse aperture, along the surface of the fingers already mentioned; and laying it in front of them, near their tips, I converted these fingers into a sort of sentient tenaculum, which, with little pain to the patient, I pushed into the back of the womb, near the fundus, and then drawing the womb downward and backward, towards the point of the os coccygis, as I carried the fingers upward and forward, I succeeded ultimately in placing the tips over the fundus in the manner of a blunt hook; after which, by a movement of retroversion, the womb was very speedily brought downwards and backwards, into the palm of the left hand, then lodging in the vagina, where, at this part of the operation, the diseased mass might be seen distinctly enough, lying just within the genital fissure.

"*Fourth Stage of the Operation.*—The process of removal being brought to this point, the diseased structure still in the palm of my hand, remained in connexion with the sides of the pelvis, by means of the fallopian tubes and broad ligaments, and with the bladder by means of the peritoneum, the front of the vagina, and interposed cellular web,—parts which were easily divided, so as to liberate the mass to be removed. The broad ligaments were cut through, close upon the sides of the uterus, and in dividing the vagina great care was taken to keep clear of the neck of the bladder and the ureters. This division of these attachments, and the removal of the diseased mass, constituted the fourth step of the operation. Some bits of indurated vagina, altogether not larger than the common bean, were left in the pelvis, to be removed at some future period, should symptoms require. This fact is worth recording.

"To this circumstantial account of the operation may be added a few remarks. The intestines did not protrude. About an ounce of blood was lost when the back of the vagina was divided, three or four more ounces following when the vagina was cut in front. Ligatures, tenacula, and forceps, were in readiness to secure the vessels, but these were not required.

"The intestines were felt at one time only, namely, when two fingers were lying out through the opening in the vagina behind. Of course some pain was felt when the first incisions were making, and when, as in ordinary obstetric operations, the hand was urged into the vagina; but the principal distress was occasioned by drawing down the uterus, when the retroversion was accomplished, and the ligaments were put upon the stretch.

"The pains and complaints scarcely exceeded those observed in instrumental deliveries. The patient lay in the ordinary obstetric position, and required no restraint. The insertion of the hook into the back of the uterus did not occasion much suffering. The operation, from first to last, occupied about an hour, but much of this time was spent in reposing and considering what might best be done. With better instruments, and greater activity, the whole operation might most probably be completed in five minutes. In obstetrics, however, celerity is considered to be in itself a secondary merit, and the operation was conducted on obstetric principles. The general range of the pulse was between 120 and 130, a frequency common in delivery by instruments.

"When the last gush of blood was observed, the pulse became imperceptible in the wrist, returning however in the course of ten or fifteen minutes. A few ounces of spirits were administered to the patient as the operation proceeded. Throughout the process the fore-finger of the left hand was the principal instrument, and the scalpels and hooks were employed merely as the means of arming the finger for its various operations. The professional friends who favoured me with their presence were Dr. Elliotson, Mr. Callaway, Mr. B. Cooper, Mr. Key, and Mr. Morgan. An accident deprived me of the presence and assistance of my friend Dr. Roots."

Five months have now elapsed since the operation and "the patient is fat and well." We have no hesitation in characterizing the foregoing operation as one of the boldest that has ever been performed on British soil.

### ADMISSION OF AIR INTO A VEIN.

It is well known that Dupuytren lost a female patient, while on the operating table, in consequence of air rushing into a wound of the external jugular vein. Some people have been sceptical as to this fact, which strongly corroborates Dr. Barry's doctrines. M. Bouley, a Parisian veterinarian, while bleeding a horse in the jugular vein, suspended, for a few seconds, the pressure below the orifice, and instantly heard a rushing noise, which he had often before heard, in the course of his practice, and therefore did not attend to it. The bleeding over, the horse was led to the stable, when the animal became quickly affected with general tremor, laborious breathing, small, irregular pulse—and, finally, fell down as if stricken with lightning. M. Bouley now conceived that the noise he had heard, was the transit of air along the vein. He instantly opened another vein, and, as the blood flowed, the animal revived. The two cases now on record should put surgeons on their guard when bleeding from the jugular vein. The orifice ought to be carefully closed before the pressure is taken off the inferior portion of the vessel.

### COMPLETE SCHOOL OF MEDICINE AT CAMBRIDGE.

The medical world will be rejoiced to learn, that Oxford and Cambridge—especially the latter, have been lately raised, by a sort of incantation, into regular and pre-eminent schools of medicine, where the sublime doctrines of Plato and conic sections are mingled with the lighter studies of pathology and clinical practice! The curricula have been promulgated in due form—and already has a disciple of the new school passed the dread ordeal of examination, compared with which the Parisian TENTAMEN is a mere song!* Only think that a *Cam bachelor* should be forced to commit to memory the devious paths of the *par vagum*—the three great branches of the cœliac artery—the use of the pericardium—and the *meaning* of the small omentum!† So much for anatomy. In respect to physiology, it is to be presumed, from the following question, that the name of one Charles Bell has actually been heard on the waters of the Cam and the Isis. "What is the difference of function *attributed* to the fifth and seventh pair?" It is clear from this, that the notion of the *seventh* pair being the nerves of audition, is quite exploded at Cambridge. As to the portio *dura*, it would be too *hard* a task for the bachelor to explore its functions.

In the second examination on pathology and therapeutics, we were delighted to observe how admirably the examinators direct the minds of their candidates into the best and most useful channels of pathological and therapeutical investigation. Was there ever such a sapient question asked, in any age or country, as the following?

"What remedy does Sydenham recommend in the diarrhœa supervening on gout?"

O thou naughty Haviland! Let us ask thee one question, and answer it without retreating to thy library. What is the first letter of the second column of the

* See Medical and Physical Journal for August 1828, p. 178, where the ordeal is detailed.

† "What is *meant* by the small omentum?"—*Ib.*

third page of the Morning Chronicle, published on Saturday, the 9th of August, 1818? Answer us that, and we will laud thy memory, whatever we may think of thy discretion.

### Oxford *versus* Cambridge.

We had scarcely finished the above reflexions, when we took up the 36th No. of the Medical Gazette, and perused, with some interest, the leading article, on "the Fellowship of the College." We will not venture to say, though certainly we *suspect*, the source of the article. It embraces a subject that divides itself into four parts—the existence of a great evil in the College—the consequences of this evil—the causes thereof —and the remedy.

1mo. The existence of the evil is thus admitted :—"The College of Physicians has certainly not acquired strength by the numerical increase of its fellows ; yet it ought to have done so. Out of a hundred men who, by this discipline, (Oxford and Cambridge study) would have enjoyed uncommon advantages of previous education, more eminent physicians must have grown up than are to be found amongst the Fellows of the College."

2do. So much for the *existence* of the evil—it is with fear and trembling we venture to contemplate the *consequences* of it. One of the consequences is so pressing, and so near at hand, that we place it first on the list. "Some plan must be quickly thought of, or the heavy *weight* which is adding itself, year after year, to the *tail* of the Fellowship, will *pull the College to the ground, and keep it there.*" Mercy on us! We had heard that a man, a ship, or an edifice, might be *top-heavy*, and thus rendered liable to tumble down; we also know that there is a certain animal called a sheep (we mean no invidious comparisons) at the Cape, whose *tail* becomes of a very inconvenient size and weight ;—but we really never dreamt that the *tails* of the Fellows grew annually at such a rate as our cotemporary has stated—much less did we imagine that, if their number were doubled, they would ever be able, with such instruments, to "pull to the ground," such a massive structure as that in Pall Mall East! This, however, is not the worst of the consequences. It is positively asserted by our cotemporary, that this increasing weight of tail in the fellowship "*will ruin the respectability of the profession.*" The writer does not suppose, of course, that the practitioners of surgery, pharmacy, or midwifery, constitute any part of the "Profession"— or that they can have any thing to do with its respectability or advancement. All depends on the Fellowship—and, strange to say, on their tails rather than on their heads !

3tio. *Causes of the Evil.* It is wonderful to observe with what rapidity error is fading away before the light of truth. The reason why the Fellows of the College have distinguished themselves more in general literature and science than in strictly medical pursuits, was generally considered to be their long residence at Oxford and Cambridge. This is now found to be an erroneous notion. The cause of their not shining in medicine is the *shortness* of their stay in the English universities ! !

"There are constantly to be found in Cambridge a number of men who would never have thought of going thither, but for the sake of seeking a way into the College of Physicians." "Nine half-terms at Cambridge complete the residence required for a medical degree." "Among these medical term-trotters at Cambridge are to be found tradesmen, and refugees from other professions, and physicians who have formerly graduated elsewhere." Such being the case, the following inference is undeniable.

"The rank of physic, as an *honorary* profession, in this country, is greatly upheld by its having constantly among its members those who have been educated in the same manner, and in the same habits, with the *best style* of English gentlemen."

This *best style* is only at Oxford, where no half-terms—or, what is the same thing, *half-gentlemen*, are allowed—and, consequently, where shining characters *only* can be formed. Now, then, the secret comes out. It is from Cambridge that the *heavy-tailed* Fellows come—the term-trotters—tradesmen's sons—refugees— and foreign graduates !

"This number is increasing every year, and if the University do not reform their system, or the College of Physicians theirs, this facility of obtaining the most honorable degree in medicine *will ruin the respectability of the profession.*"

We do not agree with the writer, (who is manifestly of the high aristocratical party) that this facility of obtaining medical degrees at Cambridge will ruin the respectability of the profession, because we conceive that the Fellows of the College form but a very diminutive proportion of that profession. But we believe, and we long ago stated our belief, that the invidious distinction between Fellows and Licentiates would, in twenty years, completely cure itself. The degradation of the Licentiate will drive the rising generation of English physicians through the prescribed Channel, and the numbers which must flock into the Fellowship, whether they have heavy or light tails, will effectually destroy the monopoly. So far from regretting this consummation, we "rejoice and are exceeding glad" at the prospect. It is one of those instances of retributive justice which every now and then refresh the eye of the philosopher, as an Oasis in the desert does that of the traveller.

4to, The Remedy. The evil which our contemporary so much deplores is, in our eyes, no evil at all; but the natural corrector of an antecedent evil. Let us hear, however, the remedy which he proposes.

"Now the whole profession are interested in the remedy of this evil, for the whole profession are concerned in the character of the body which presides over it. But what shall be the remedy? Probably, the universities would not bear patiently the interference of the College in their discipline, or even allow it to suggest to them the condition of granting medical degrees. But the College might do this—it might cease to examine the bearers of English degrees for admission into the Fellowship, as a matter of course; it might examine them simply for a license, and elect them subsequently according to their character, with or without a second examination, into the Fellowship." 309.

Much as we object to the mode in which the Fellowship is now obtained, we object still more strongly to the one proposed above. By this new plan the College would be emancipated from all laws, even of its own construction—except one—sic volo, sic jubeo! The election to the Fellowship would depend on caprice, favour, intrigue,—and that body would thus be erected into one of the most despotic tyrannies that ever existed on the face of the earth! To the "tradesmen's sons"—the "term-trotters" of Cambridge—the graduates of other universities—to all but "the best style of English gentlemen," the College would be hermetically sealed! Fortunately, such a monstrous proposal of sacrificing talent, science, and learning, at the shrine of aristocratic wealth, would be scouted in the present day—for the time *has* come, when respectability in medicine is the result of moral worth and professional talent;—not to be put on with a black-silk gown, after a certain period of residence on the banks of the Isis.

The article which has given rise to these observations, and which evidently emanates from the *high aristocracy* of the College, is not one of the least significant "signs of the times." There is a tolerably old maxim that—"a house divided against itself cannot stand." That the aristocratic Oxonians, (the Absolutists) eye with jealousy the semi-plebeian Cantabs, (or Liberals) is now beyond a doubt. It remains to be proved whether the *heavy heads* of the former, or the *heavy tails* of the latter shall prevail. If an unbiassed and independent spectator may be allowed to form an opinion, we would say that the *tails* will gain the day—for if there is to be a dead weight about a body of men, let it, in God's name, be located near the bottom rather than the top of that body.

We have only to reiterate a piece of advice which we, some years ago, gave to those parents who were educating their sons as physicians in this country. By the regulations of Cambridge, nine half-terms, of six weeks each, and spread over three years, qualify for a degree, and secure the admission, in due time, to the Fellowship. Edinburgh requires four years. Now we fearlessly maintain, that these four years, spent in London, and keeping terms at Cambridge, will insure a better medical education than Edinburgh or Oxford can afford—will not be more expensive than the former—and will save the Edinburgh graduate from the mark of degradation which, as a licentiate, he must undergo, if he settles in the metropolis, or within seven miles thereof. This information should be pondered on by parents!

---

SMOKING TOBACCO IN HERNIA.

In a recent journal of this metropolis, there is a serio-ludicrous proposal from Mr. Wallace, of the Royal Navy, respecting the superiority of smoking tobacco over the same drug used as an enema. It is probable that Mr. Wallace has not accurately observed the effects of tobacco given per anum. The death-like coldness and pallor, the feeble pulse, the relaxation of the muscles, and the tendency to syncope, are independent of sickness or vomiting, and precede that operation. These phenomena are totally different from that nausea which is produced by smoking tobacco for the first time. We can assure Mr. Wallace that his plan would be entirely fruitless, and would, in no efficient degree, contribute to the reduction of a hernial protrusion.

---

### HOSPITAL PRACTICE.

### 61. ROYAL INFIRMARY OF GLASGOW.

A quarterly report of the cases in the surgical wards of the above institution, is published by Dr. Anderson, in the third Number of the Glasgow Journal. Before he proceeds to particular cases, the Doctor has given a table of maladies admitted, and operations performed, shewing, in the gross, the surgical practice of the hospital.

### I. ARTIFICIAL ANUS TREATED BY COMPRESSION AND THE CAUTERY.

Baron Dupuytren's memoir, of which we have given so ample an account, will make, if we mistake not, an important improvement in the cases of artificial anus occurring in consequence of hernia or wounds. The subject of the present case was a Mary Stewart, admitted on the 10th November, with a very large sore in the left groin, extending from the spine of the ilium to the symphysis pubis. It was three inches broad, passed above Poupart's ligament, was ragged, irregular and sloughy, and a sinus extended from it down by the side of the labium, from which could be pressed a good deal of purulent matter. The gut protruded as a round firm substance in the centre of the sore, and immediately above the protrusion was seated the opening, through which all the fæces were evacuated. The poor girl was hectic and greatly emaciated, and the pulse was so frequent and feeble as not to admit of being counted. For twelve months she had been subject to femoral hernia, which, a month before admission, had apparently got strangulated, and sloughed in eight days, from which time the fæces had passed by the wound.

"This case was very unpromising, and from the excessive debility, it seemed almost hopeless. A nourishing diet, with a liberal allowance of wine and brandy, produced little improvement. The appearance of the discharges soon showed that her debility chiefly arose from inanition, owing to the artificial opening having taken place in a part of the gut where the process of assimilation was still incomplete. With the double purpose, therefore, of affording nourishment, and dilating the lower intestines, I directed the use of large beef-tea enemata three times a-day.

"The next object was to prevent the escape of the contents of the gut, which not only kept up the debility, but produced sloughing of the sore, and hindered it from healing. The aperture admitted the finger, by which it was ascertained that only one side of the gut had been destroyed, and that the other side formed a continuous surface towards the abdomen. This case, therefore, did not require the application of the ingenious instrument of Baron Dupuytren. Plugging the opening with various conical shaped sponges was first tried; but she could not bear the degree of pressure necessary to retain them *in situ*. Recourse was therefore had to a long cylindrical tent of lint firmly rolled up.
